# Earth’s east–west albedo symmetry

**DOI:** 10.1038/s41586-026-10624-2

**Published:** 2026-06-03

**Authors:** Jianhao Zhang, Jake J. Gristey, Graham Feingold

**Affiliations:** 1 Cooperative Institute for Research in Environmental Sciences (CIRES), University of Colorado Boulder, Boulder, CO USA; 2 Chemical Sciences Laboratory, National Oceanic and Atmospheric Administration (NOAA), Boulder, CO USA; 3 Laboratory for Atmospheric and Space Physics (LASP), University of Colorado Boulder, Boulder, CO USA

**Keywords:** Atmospheric dynamics, Climate and Earth system modelling, Projection and prediction

## Abstract

Earth’s albedo is fundamental to the planetary energy budget^1^. The Northern Hemisphere (NH) and Southern Hemisphere (SH) contribute essentially equally to the planetary albedo—a remarkable yet puzzling phenomenon known as hemispheric albedo symmetry^1–6^. Although such symmetry is rare, it is not unique^7^. Nevertheless, other symmetry pairs have remained unexplored, despite their potential to illuminate possible causes of albedo symmetries and implications for the planetary energy budget. Using a 25-year satellite record, here we show that Earth also exhibits a unique and persistent east–west (E–W) albedo symmetry: the 27° E meridian divides the planet into an Eastern Hemisphere (EH) and a Western Hemisphere (WH) that reflect nearly identical amounts of sunlight. In contrast to the NH–SH symmetry, the EH–WH symmetry encapsulates a distinctive ‘triple symmetry’ in which clear-sky albedo, cloud radiative effect and open-ocean fraction all exhibit hemispheric symmetry around this meridian. This EH–WH symmetry arises from greater high-cloud reflection in the EH balancing greater low-cloud reflection in the WH. Furthermore, interannual variability in the EH–WH symmetry tracks the phase of the El Niño–Southern Oscillation (ENSO), indicating a potential connection to general circulation. This discovery of the EH–WH albedo symmetry and its emergence as a triple symmetry provides a reduced degree-of-freedom constraint for Earth system models (ESMs) and stresses the critical nature of continued Earth radiation budget observations under a rapidly changing climate.

## Abstract (web summary)

Analysis of a 25-year satellite record shows that Earth has a persistent east–west albedo symmetry split at 27° E, with clear-sky albedo, cloud radiative effect and open-ocean fraction exhibiting a triple symmetry around this meridian.

## Main

The Earth reflects about 29% of the incoming solar radiation back to space, an intrinsic property of a planet known as the planetary albedo^1^. An intriguing observation has emerged since spaceborne monitoring of Earth’s radiative fluxes became available half a century ago: the NH and SH reflect almost identical amounts of sunlight^1–4,7–9^. Although this symmetry is known to arise from a cloudier SH balancing more reflective clear skies in the NH^1,5,8,10^, the lack of satisfactory mechanistic explanations for how the north–south (N–S) symmetry is maintained leaves open the possibility of a coincidence, and the N–S symmetry remains a mystery^6,8^. This possibility is further fuelled by the fact that observations may now suggest a departure from N–S symmetry^11–13^.

Given that the Earth is approximately spherical, it is unsurprising that one can divide it into two non-overlapping hemispheres that reflect equal amounts of sunlight. However, the chance of achieving such a hemispheric symmetry within 0.1 W m^−2^ is less than 3% (ref. ^7^), making the hemispheric symmetry unlikely to be coincidental. An unexplored question is what we can learn from these rare hemispheric pairs in which symmetry holds, insights that could be fundamental to understanding the Earth’s climate system. Similarities or differences across symmetry pairs could shed light on the underlying couplings among Earth system components.

This work presents the first investigation of the Earth’s hemispheric albedo symmetry as a function of longitude. We divide the planet into EHs and WHs at every longitudinal great circle, with one-degree increments, and identify 27° E (with 153° W completing the great circle) as the unique meridian that produces an E–W albedo symmetry using 25 years of satellite-measured top-of-atmosphere (TOA) shortwave (SW) fluxes. The finding of the E–W symmetry at 27° E is notable for its persistence and the fact that it represents the only longitudinal divide yielding such balance. Moreover, it coincides with the meridian that separates the Earth into two hemispheres containing almost the same amounts of ice-free ocean. Put differently, the ratio of land to ocean correlates strongly with the reflected solar flux in the two hemispheres, a feature in strong contrast to the N–S symmetry. In a hypothetically cloud-free world, this correspondence might not be surprising, given the contrast in surface reflectivity between land and ice-free ocean. However, for the cloudy planet we inhabit, the persistence of this symmetry becomes nontrivial, underscoring the need to understand the role of clouds in maintaining the observed E–W symmetry.

Using more than two decades of Clouds and the Earth’s Radiant Energy System (CERES) observations^14–17^, we show that the E–W symmetry at 27° E arises from a high-cloud-dominated EH balancing a low-cloud-dominated WH, and from an EH with more reflective oceanic and ice-covered regions balancing a WH with more reflective ice-free continental regions. This E–W symmetry, coinciding with an even divide of hemispheric ice-free-ocean fractions and a near symmetry in cloud radiative effect (CRE)—a triple symmetry—is not captured by climate models. We find a strong correlation between the interannual variability of the E–W hemispheric albedo symmetry and the phase of ENSO, suggesting that large-scale atmospheric circulation, particularly the Walker circulation, may play an important role in maintaining this symmetry.

## Observed triple symmetry at 27° E

**Fig. 1:**
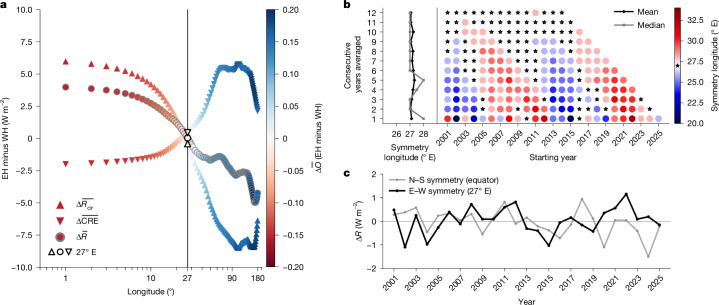
Demonstration of Earth’s E–W hemispheric albedo symmetry and its persistence. **a**, 25-year mean differences in reflected SW radiation based on CERES EBAF^51^ as a function of longitude and coloured by the difference in ice-free-ocean fraction ($\Delta \overline{O}$) between the EH and the WH (EH minus WH). All-sky reflection difference ($\Delta \overline{R}$) is shown by circles, clear-sky reflection difference ($\Delta \overline{{R}_{{\rm{clr}}}}$) is shown by upward triangles and CRE difference ($\Delta \overline{{\rm{CRE}}}$) is shown by downward triangles. Values at 27° E are highlighted with black outlines. An approximate triple symmetry at 27° E, for $\overline{O}$, $\overline{{R}_{{\rm{clr}}}}$ and $\overline{{\rm{CRE}}}$, is a unique feature of the E–W symmetry, distinct from the N–S symmetry. **b**, Identified E–W $\overline{R}$ symmetries as a function of consecutive years averaged (from 1 to 12 years on the vertical axis) and a function of starting year for the averaging (on the horizontal axis). The longitudes of the identified E–W symmetries are shown by colours, with 27° E denoted as a star, with means and medians shown on the left as a function of consecutive years averaged. The E–W symmetry is robustly identified at 27° E in the EBAF record at decadal timescale. An oscillating pattern at the averaging scale between 1 and 10 years is evident, hinting at ENSO variability. **c**, Interannual variability of the E–W albedo symmetry at 27° E (black), overlaid with the N–S albedo symmetry (grey) for comparison. The E–W and N–S symmetries have similar magnitudes of interannual variability.

Figure 1 illustrates the hemispheric difference in CERES-observed TOA SW reflection as a function of longitude, revealing the difference in all-sky SW reflection ($\overline{R}$, overbar denoting climatology) between the two hemispheres to be approximately monotonic functions of longitude and of ice-free-ocean fraction ($\overline{O}$). A hemispheric symmetry in $\overline{R}$ is identified at 27° E ($\Delta \overline{R}$ of 0.04 ± 0.24 W m^−2^) based on the 25-year (2001–2025, inclusive) Energy Balanced and Filled (EBAF) record (Methods). Therefore, we henceforth use 27° E as the reference meridian for the EH and the WH and for assessing the degree of asymmetry in interannual variabilities and in climate models.

The monotonic relationship between $\Delta \overline{R}$ and $\Delta \overline{O}$ might have been straightforward to understand in a world without clouds, given the strong contrast in SW reflectivity between land and ice-free ocean. Indeed, a tight relationship between $\Delta \overline{{R}_{{\rm{clr}}}}$ and $\Delta \overline{O}$ is observed, with the symmetry also located near 27° E (Fig. 1a, upward triangles). The co-located symmetries of $\overline{R}$ and $\overline{{R}_{{\rm{clr}}}}$ lead to a new and important finding: the hemispheric difference in CRE ($\Delta \overline{{\rm{CRE}}}$, downward triangles) is also near zero at this meridian. Together, symmetries in *R*_clr_ and CRE both emerge at approximately 27° E, where the ice-free-ocean fraction is almost identical between the two hemispheres, revealing a unique triple symmetry (±0.5 W m^−2^; Fig. 1a) that is distinct from the N–S albedo symmetry.

Unlike the N–S symmetry in which the equator is the apparent and only divide for the two hemispheres, there is no such predefined divide for the E–W symmetry. The question, then, becomes: how robust is the E–W symmetry at 27° E and is it persistent over time? To address this question, we identify the E–W albedo symmetry as a function of consecutive year(s) being averaged using the 2001–2025 (inclusive) EBAF record (Fig. 1b). For 1-year averaging, the identified E–W symmetries span about 10° in longitude between 20 and 30° E (coloured circles). Beyond 1-year averaging but shorter than 10-year averaging, an oscillating pattern around 27° E is evident, hinting at an interannual variability in E–W symmetry that might be tied to some multiyear oscillation phenomena (for example, ENSO; discussed later). At the decadal timescale that encompasses both phases of this oscillation, the E–W symmetry is robustly identified at 27° E. Although variability persists for averaging periods shorter than 10 years, the mean and median are consistently confined between 27° E and 28° E (Fig. 1b). We observe similar magnitudes of interannual variability in Δ*R* between the E–W symmetry (at 27° E; ±0.24 W m^−2^) and the N–S symmetry (at the equator; ±0.20 W m^−2^)^8,9^ (Fig. 1c).

## Clouds compensate to sustain symmetry

**Fig. 2:**
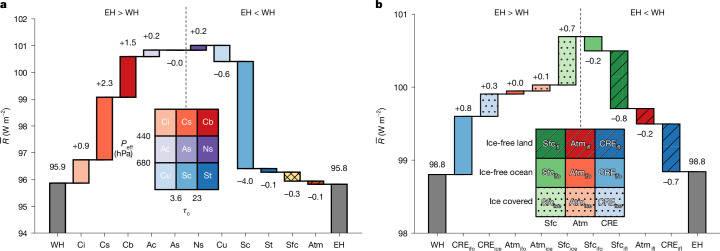
Hemispheric compensations across components of *R* sustain the E–W symmetry. **a**, Waterfall chart showing hemispheric compensations across components of *R* (coloured bars), with positive (negative) values of the coloured bars denoting greater EH (WH) reflection. EH and WH are demarcated at 27° E (see main text). *R* is decomposed into contributions from surface (Sfc) under clear-sky, atmosphere (Atm) under clear-sky and the CRE for different cloud types (illustrated by the inset), based on the CERES FBCT product^52^ for 2003–2022 (inclusive). Grey bars indicate the hemispheric-mean *R* ($\overline{R}$), which is nearly identical between the EH and the WH. A clear compensation between the low-level clouds (more reflective in the WH) and the high-level clouds (more reflective in the EH) is evident. **b**, As in **a** but here *R* is decomposed on the basis of surface types (using the NSIDC snow/ice coverage product^53^), including ice-free land (ifl), ice-free ocean (ifo) and ice-covered (ice) areas, and reflection components, including clear-sky atmosphere (Atm), clear-sky surface (Sfc) and CRE (illustrated by the inset; Methods). The CERES EBAF record^51^ for 2001–2025, inclusive, is used for this decomposition. The approximately 3 W m^−2^ difference in hemispheric $\overline{R}$ between the two CERES products reflects differences in how monthly mean fluxes are derived (Methods). Through this lens, the E–W symmetry is maintained by greater reflection from the ice-free continental regions in the WH compensating for greater reflection from the ice-covered and ice-free oceanic regions in the EH.

**Extended Data Fig. 1:**
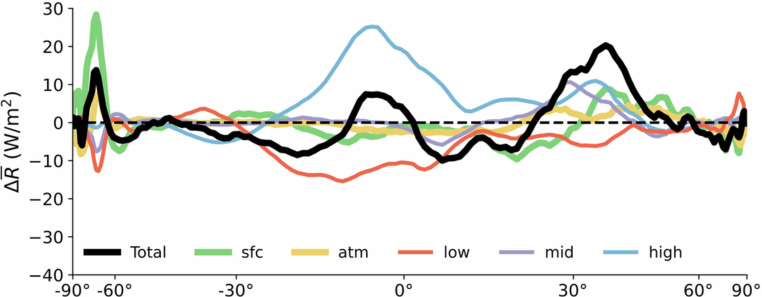
E–W albedo symmetry from compensating high-cloud and low-cloud reflection. Climatology of zonal-mean hemispheric difference (defined at 27° E) in *R* and its components, including clear-sky surface (sfc), clear-sky atmosphere (atm), low clouds, mid clouds and high clouds, using the CERES FBCT^52^ dataset for 2003–2022 (inclusive).

The hemispheric symmetry in CRE at 27° E is a surprising finding because all three of Earth’s semipermanent, extensive stratocumulus decks reside in the WH, over subtropical oceans off the coasts of California, Chile and Namibia^18^. This implies that other cloud components must be more reflective in the EH than in the WH to compensate for the bright stratocumulus decks in the WH. To examine compensations among components of the Earth system that help maintain the E–W albedo symmetry, we first decompose CRE into contributions from different cloud types^6^ and *R*_clr_ into surface and atmospheric contributions^1,19^ (*R*_sfc_ and *R*_atm_; Methods). This analysis reveals that greater reflection from high-level clouds in the EH is balanced by greater reflection from low-level clouds in the WH. This balancing is largely responsible for maintaining the E–W symmetry, as hemispheric differences in the other components remain relatively small and compensate among each other (Fig. 2a). The hemispheric compensation between low and high clouds is also evident in the zonal mean (Extended Data Fig. 1); greater EH reflections from high clouds peak in the deep tropics, whereas greater WH reflections from low clouds extend from the tropics into the subtropics.

**Extended Data Fig. 2:**
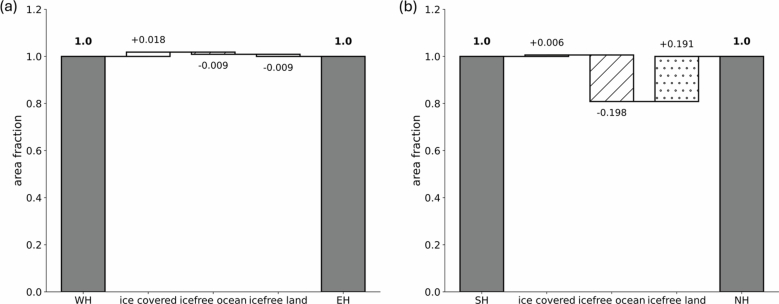
Surface type contrasts are minimal in E–W but pronounced in N–S. Hemispheric difference (defined at 27° E) in area fractions for ice-covered regions, ice-free oceans and ice-free lands, between the EH and the WH (**a**) and between the NH and the SH (**b**). The NSIDC snow/ice coverage product^53^ (2001–2025, inclusive) is used.

**Extended Data Fig. 3:**
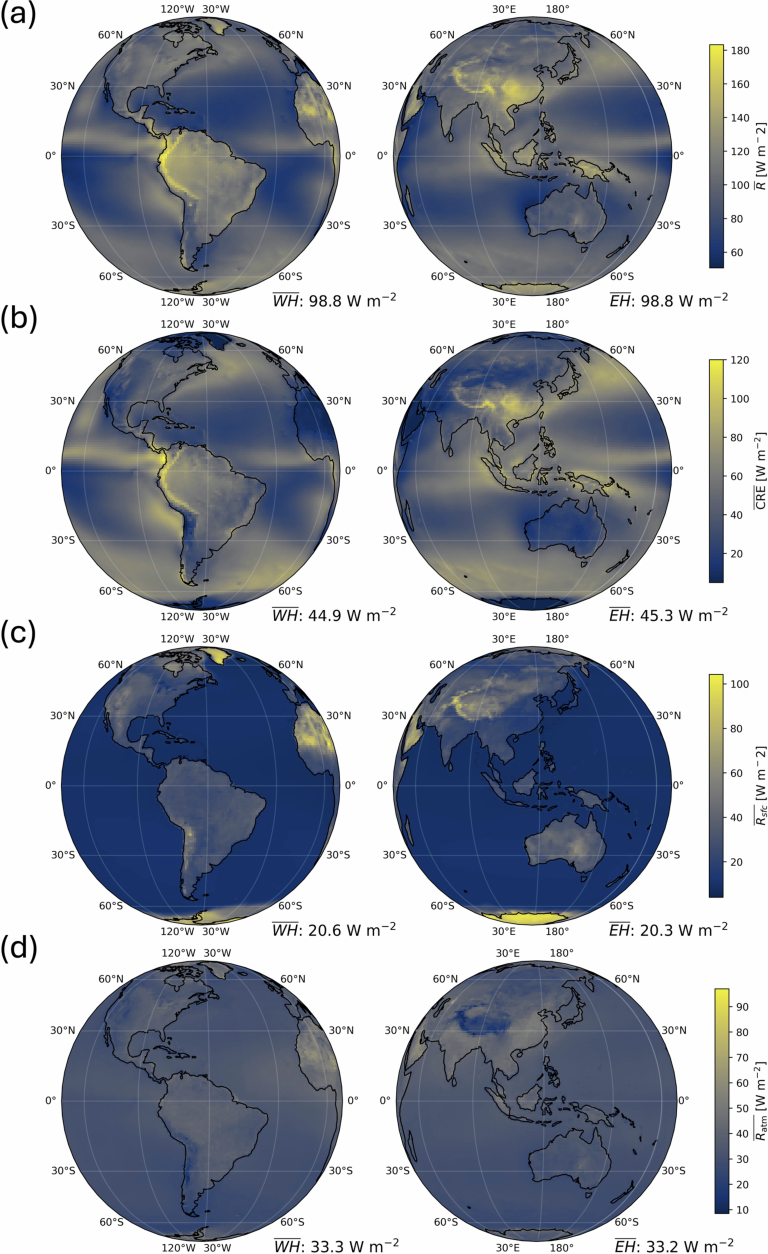
Cloud radiative effect dominates spatial variability of *R.* Climatological maps of hemispheric mean reflection for *R* (**a**), CRE (**b**), *R*_sfc_ (**c**) and *R*_atm_ (**d**) for the WH (left column) and the EH (right column), dividing at 27° E. The CERES EBAF^51^ 2001–2025 (inclusive) record is used. In the EH, the greater reflection over oceanic and ice-covered regions arises mainly from the broad coverage of Southern Ocean storm tracks and the expansive anvil clouds associated with tropical deep convections over the Maritime Continent. In the WH, this is compensated by greater reflection over ice-free continental regions. A substantial part of this reflection comes from clear-sky surface albedo, associated with high-elevation terrain such as the Rockies and Andes, as well as bright deserts and arid regions, including the Sahara, Namib, the southwestern United States, Mexico, Atacama and Patagonia. The other main contributor is continental CRE, arising mainly from orographic clouds along the Andes and the vast ‘sea’ of cumulus clouds over the Amazon rainforest.

A unique feature of the E–W symmetry is the near-identical ice-free-ocean fraction in the two hemispheres (Fig. 1). This naturally raises a question: is this ocean–land ratio symmetry a mere coincidence or is it essential to maintaining the observed E–W albedo symmetry? To address this question, we further separate TOA SW reflections into ice-free land (ifl), ice-free ocean (ifo) and ice-covered (ice) regions, based on time-varying, satellite-retrieved surface type information at each grid location, on top of the surface (Sfc), atmosphere (Atm) and CRE decomposition (Methods). Figure 2b shows the hemispheric differences of the nine components of *R* resulting from this decomposition. A compensation between land columns and ocean and ice columns is observed, yielding the hemispheric symmetry. This is particularly intriguing because, unlike the pronounced N–S contrast in land–ocean fractions, the area fractions of ice-free ocean, ice-free land and ice-covered regions are nearly identical between the EH and the WH (Extended Data Fig. 2). Therefore, the E–W symmetry is essentially maintained through compensation between the more reflective ice-free continental regions in the WH and the more reflective oceanic and ice-covered regions in the EH. A discussion on the geographical features of each hemisphere to help dissect this hemispheric compensation is provided in Extended Data Fig. 3 and its caption.

**Extended Data Fig. 4:**
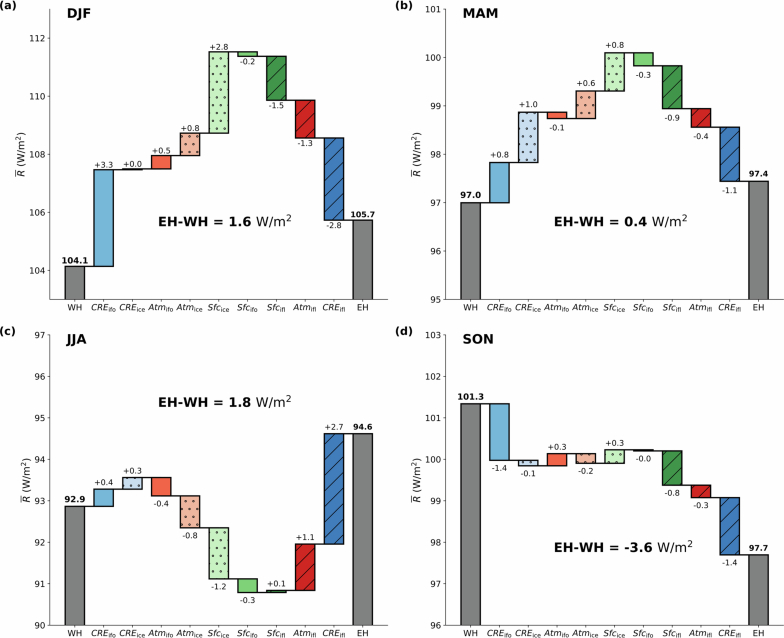
The E–W symmetry breaks down at seasonal timescales. Waterfall charts showing hemispheric compensations across components of *R* (coloured bars), with positive (negative) values of the coloured bars denoting greater EH (WH) reflection. EH and WH are demarcated at 27° E. Components of *R* include cloud radiative effect (CRE), clear-sky atmosphere (Atm) and clear-sky surface (Sfc), partitioned over ice-free land (ifl), ice-free ocean (ifo) and ice-covered (ice) regions. Panels show different seasons: December–February (DJF; **a**), March–May (MAM; **b**), June–August (JJA; **c**) and September–November (SON; **d**). Hemispheric difference in $\overline{R}$ between the EH and the WH are labelled for each season. The CERES EBAF^51^ 2001–2025 (inclusive) record is used.

Another distinct feature of the E–W symmetry is that, unlike the N–S symmetry, it does not require a full annual cycle to achieve an equal amount of SW reflection from the two hemispheres, given that the Earth rotates in the E–W direction. We found that, at seasonal timescales, the E–W symmetry and the compensation across its components generally break down, mainly because of the strong regional–seasonal variability in CRE (Extended Data Fig. 4). The hemispheric differences in CRE at seasonal timescales range from +3.5 W m^−2^ (June–August) to −2.9 W m^−2^ (September–November). We note, however, that the ocean/ice–land compensation pattern is largely maintained during boreal winter and spring.

**Extended Data Fig. 5:**
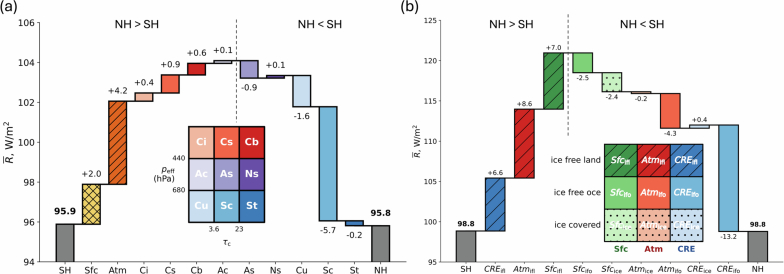
N–S albedo symmetry from compensating clear-sky and cloud reflection. Waterfall charts showing hemispheric compensations across components of *R* (coloured bars), with positive (negative) values of the coloured bars denoting greater NH (SH) reflection. For **a**, *R* is decomposed into clear-sky surface (Sfc), clear-sky atmosphere (Atm) and cloud radiative effect (CRE) for different cloud types (illustrated by the inset), based on the CERES FBCT^52^ product for 2003–2022 (inclusive). For **b**, components of *R* include cloud radiative effect (CRE), clear-sky atmosphere (Atm) and clear-sky surface (Sfc), partitioned over ice-free land (ifl), ice-free ocean (ifo) and ice-covered (ice) regions, using the 2001–2025 EBAF^51^ record. We note that panel **a** is similar to Fig. 1a of ref. ^6^, as we follow the same plotting convention. The slight difference in hemispheric mean *R* reflects the different analysis period in ref. ^6^, from June 2002 to July 2022.

A similar compensation among different surface types is found for the N–S symmetry (Extended Data Fig. 5b) but it is much larger in magnitude and arises mainly from the strong land–ocean contrast between the NH and the SH (Extended Data Fig. 2b). Consequently, the N–S symmetry is maintained largely by stronger CRE in the SH (mostly from low-level clouds) compensating for stronger clear-sky reflections in the NH (Extended Data Fig. 5a and ref. ^6^). Thus, although TOA all-sky SW reflection is climatologically split equally between the two hemispheres, decomposition into reflection components reveals that the N–S and E–W symmetries are maintained through very different hemispheric compensations. Moreover, the corresponding hemispheric contrasts in clear-sky reflection, CRE and land–ocean fractions at these all-sky reflection symmetries also differ substantially (Fig. 1a and Extended Data Fig. 5), suggesting that the two albedo symmetries are probably not directly connected. Given the record global mean temperatures of the past few years, the question arises: are the distinct compensations that maintain the N–S and E–W symmetries robust to emerging changes in the Earth system?

## Recent trends towards asymmetry

**Fig. 3:**
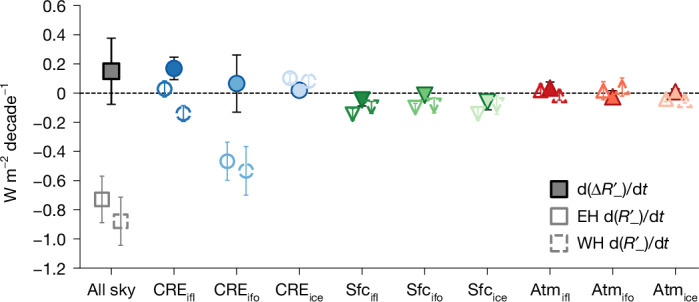
Cloud radiative effect drives E–W asymmetry trends. 25-year (2001–2025) trends in Δ*R* (EH minus WH at 27° E) and its components are shown as filled, coloured markers. Components of *R* include CRE, clear-sky atmosphere (Atm) and clear-sky surface (Sfc), partitioned over ice-free land (ifl), ice-free ocean (ifo) and ice-covered (ice) regions. Trends in hemispheric *R* are shown as open, coloured markers, with solid and dashed outlines denoting the EH and the WH, respectively. Error bars indicate the 95% confidence level; in some cases, they are smaller than the markers. Hemispheric differences in CRE trends are largely responsible for a 0.15 ± 0.23 W m^−2^ decade^−1^ insignificant trend in E–W Δ*R*.

**Extended Data Fig. 6:**
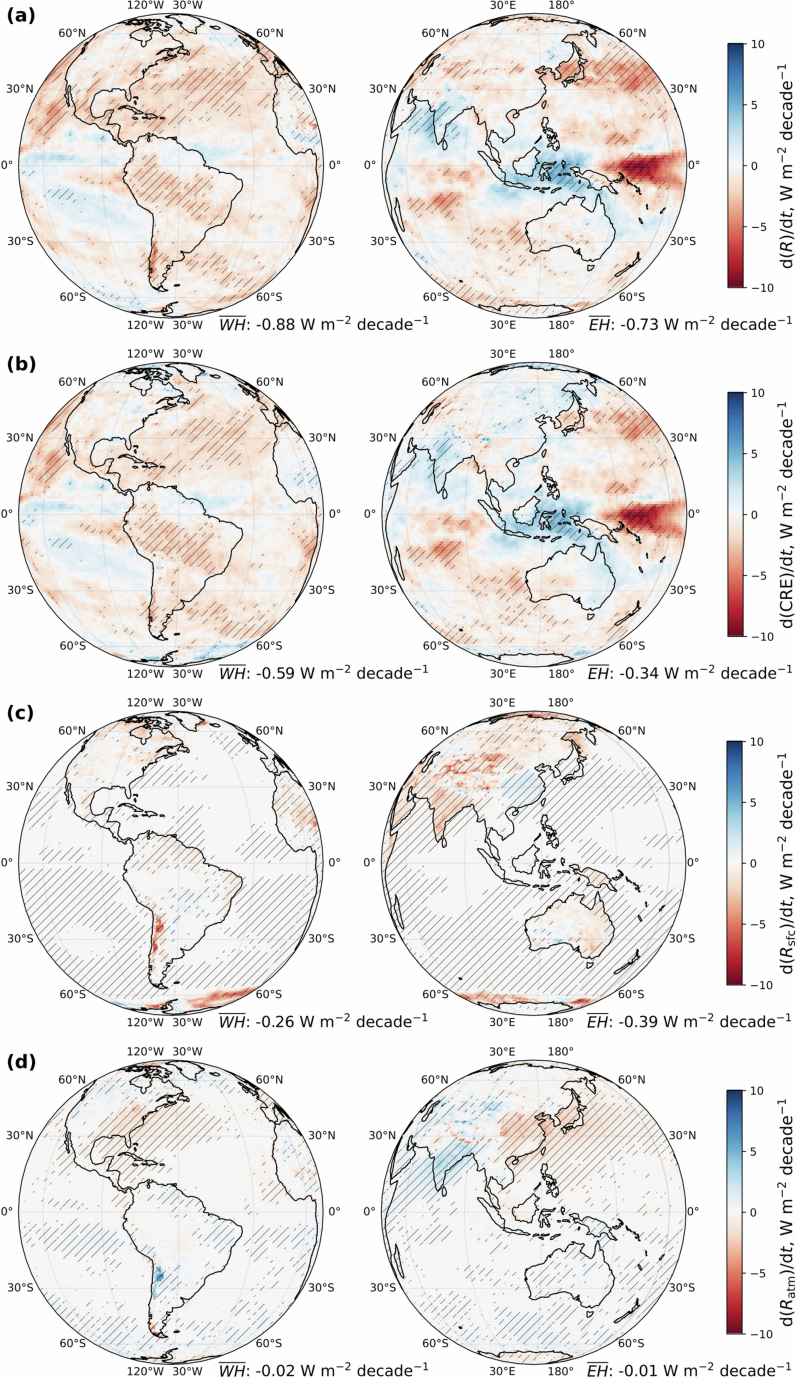
Cloud radiative effect dominates all-sky SW reflection trends. Maps of hemispheric trends in *R* (**a**), CRE (**b**), *R*_sfc_ (**c**) and *R*_atm_ (**d**). Hemispheric mean trends are labelled. Hatching denotes statistically significant trends (95% confidence). The CERES EBAF^51^ 2001–2025 (inclusive) record is used.

Changes to the Earth system over the past few decades, including pronounced shifts in sea-ice extent, forest cover, emissions and wildfire activity, pose an opportunity to test the argument that Earth’s albedo symmetries are sustained by an underlying mechanism. Figure 3 shows the 25-year (2001–2025, inclusive) trends in the E–W hemispheric Δ*R* and its components with uncertainties (95% confidence; Methods). Both hemispheres have experienced pronounced darkening trends over the past two decades, with the WH darkening more rapidly than the EH (grey open markers in Fig. 3 and maps in Extended Data Fig. 6). Although the hemispheric darkening trends are statistically significant, the trend in their difference (that is, the E–W asymmetry) remains insignificant (that is, error bars denoting 95% confidence crossing the zero line). It is clear that the overall darkening trend arises from changes in CRE globally (blue open markers), with pronounced warming effects from marine clouds. Cloud brightening over polar regions does marginally offset the marine cloud darkening, consistent with the observed correlation between retreating sea-ice extent and increasing polar cloudiness^20^. Also contributing to this global darkening is the weak, yet statistically significant, darkening of clear-sky surface reflection (green open markers). This surface darkening arises from Arctic and Antarctic sea ice loss^21,22^, agriculture-driven and forestation-driven ‘greening’ over India and western China^23^ and a global darkening of the ice-free ocean surface^24^. Overall, hemispheric-mean trends in clear-sky atmospheric reflection (red open markers) are weak and statistically insignificant in both hemispheres, owing to offsetting aerosol emission trends over China and India in the EH and to compensation in the WH between decreasing aerosol over the western Atlantic and clear-sky brightening over the Southern Ocean^13^.

**Extended Data Fig. 7:**
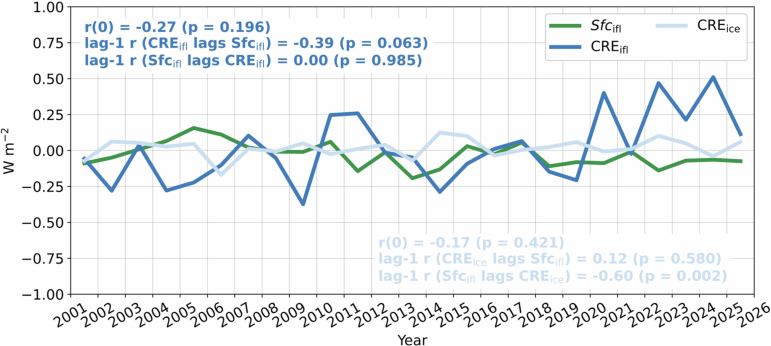
Interannual covariability between ice-covered cloud radiative effect and ice-free land clear-sky reflection. Time series of interannual variability in E–W Δ*R*_sfc_ (ifl) and ΔCRE (ifl and ice). Correlation coefficients and *P*-values evaluated at lag(−1year), lag(0year) and lag(1year) are labelled. Locally, Sfc_ifl_ negatively leads CRE_ifl_ by one year (*P* < 0.1), suggesting a lagged coupling between changes in land surface and cloud properties at interannual timescales. Such mechanistic responses of clouds to surface variations are known to exist^82–84^; one prominent example emerges over the Amazon rainforest, in which deforestation-driven surface brightening is associated with pronounced cloud darkening (Extended Data Fig. 6). Furthermore, a significant negative lag-0 correlation (*P* < 0.05; not shown) is found between CRE and clear-sky surface reflection over ice-covered regions, consistent with the known coupling between sea ice (or ice sheet) extent and polar cloudiness^20^. Notably, clear-sky surface reflection over ice-free lands (Sfc_ifl_) lags changes in CRE over polar regions (CRE_ice_) by one year with a significant negative correlation (*P* < 0.01). This points to a potential teleconnection linking emerging high-latitude changes to lower-latitude continental regions on interannual timescales. Such a connection may arise from hydrological adjustments owing to changes in oceanic and atmospheric overturning circulations in response to regional redistribution of energy^85–87^.

The hemispheric difference in the darkening trend that pulls apart the E–W symmetry at 27° E is again driven by differences in CRE trends between the EH and the WH (blue filled markers in Fig. 3 and maps in Extended Data Fig. 6). The dominant contribution is from ice-free continental regions, in which significant cloud darkening over the Amazon rainforest in the WH contrasts with offsetting, insignificant CRE brightening and darkening trends in the EH. A secondary contribution arises from expansive cloud darkening over the major stratocumulus decks, which lie predominantly in the WH^25^. This E–W asymmetry trend driven by the CRE is only marginally offset by slightly faster darkening of clear-sky surface reflection over continental and ice-covered regions in the EH (green markers). Although weak in magnitude, these offsetting trends in CRE and clear-sky surface reflection hint at coupling between the land surface and clouds that may help sustain the E–W hemispheric symmetry over longer timescales. A lagged correlation analysis based on interannual variabilities in Δ*R* and its components (Extended Data Fig. 7) has identified several potential couplings for future exploration.

**Extended Data Fig. 8:**
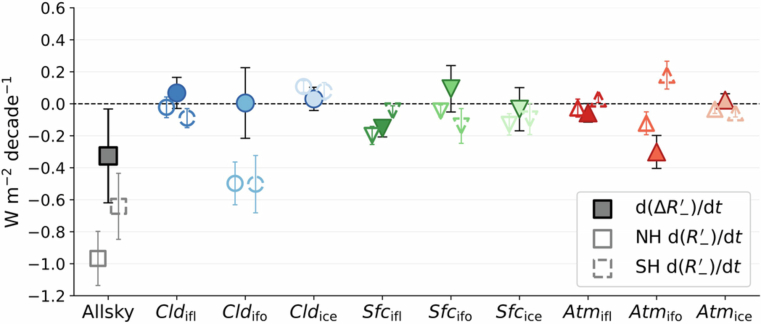
Hemispheric differences in clear-sky reflection trends lead to a departure from the N–S albedo symmetry. 25-year trends in Δ*R* (NH minus SH) and its components are shown as filled, coloured markers. Components of *R* include CRE (Cld), clear-sky atmosphere (Atm) and clear-sky surface (Sfc), partitioned over ice-free land (ifl), ice-free ocean (ifo) and ice-covered (ice) regions. Trends in hemispheric *R* are shown as open, coloured markers, with solid and dashed outlines denoting the NH and the SH, respectively. Error bars indicate the 95% confidence level; in some cases, they are smaller than the markers. The CERES EBAF^51^ 2001–2025 (inclusive) record is used to calculate the trends. Hemispheric differences in clear-sky reflection (Sfc and Atm) trends are largely responsible for a −0.33 ± 0.29 W m^−2^ decade^−1^ trend in N–S Δ*R*, indicating a statistically significant departure from the N–S symmetry.

The N–S contrast in these emerging Earth system changes over the past two decades (Extended Data Fig. 8) is expressed primarily through clear-sky atmospheric reflection: darkening in the NH from anthropogenic aerosol clean-up and brightening in the SH from enhanced natural aerosol production over the Southern Ocean^13^. Consequently, the N–S asymmetry trend derived from the 2001–2025 EBAF record is statistically significantly different from zero (grey filled marker and error bar), suggesting a breakdown of the N–S symmetry^11^. Although we cannot rule out the possibility that both symmetries may be transient features of the present climate system, the E–W symmetry is shown to be more resilient to emerging environmental changes. Notably, despite the continuation of record-breaking global mean temperatures, the N–S Δ*R* shifts back towards symmetry in 2025 (Fig. 1c). The cause of this reversal warrants further investigation, potentially involving outgoing longwave radiation^11,12^.

## Models do not capture the 27° E symmetry

The E–W albedo symmetry at 27° E offers a unique opportunity to evaluate how ESMs represent components of the Earth system, especially given the triple-symmetry feature that is absent from the N–S symmetry. In turn, model simulations of past and future climates can be used to examine the persistence of this E–W hemispheric symmetry, outside of the observational record. To do this, we analyse outputs from eight coupled ESMs that participated in the Coupled Model Intercomparison Project Phase 6 (CMIP6)^26^, the Scenario Model Intercomparison Project (ScenarioMIP)^27^ and the Geoengineering Model Intercomparison Project Phase 6 (GeoMIP6)^28^. The rationale for model selection is to capture variability in both model architecture and climate scenarios (Methods).

**Fig. 4:**
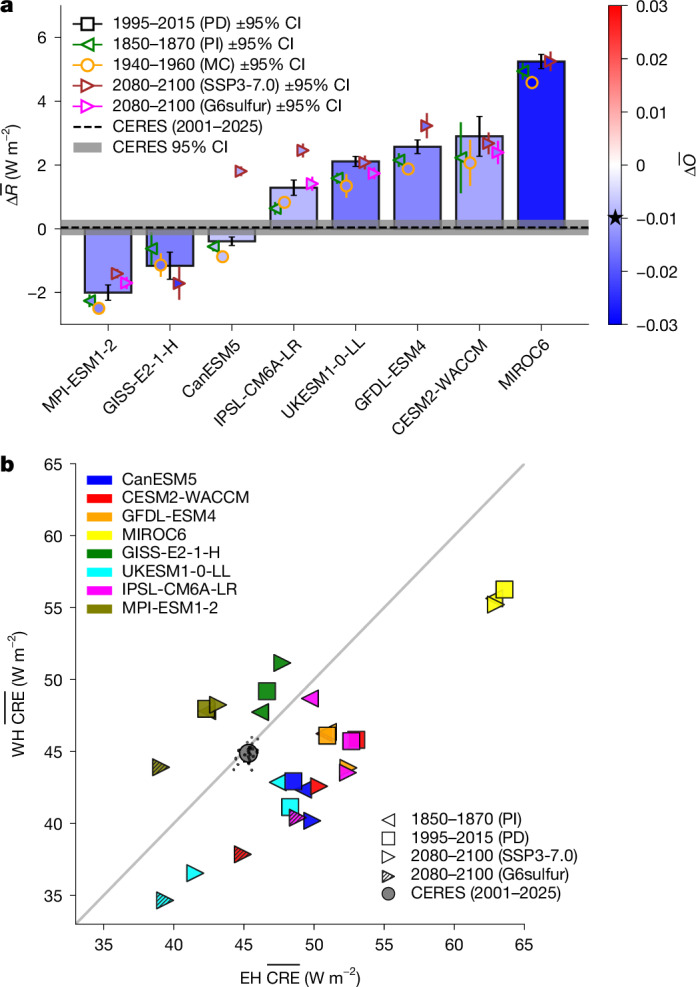
CMIP6 models struggle to reproduce the observed E–W albedo symmetry and its triple-symmetry feature. **a**, $\Delta \overline{R}$ at observed E–W symmetry (27° E) in eight ESMs that participated in CMIP6, ScenarioMIP and GeoMIP6. Present-day (PD, 1995–2015) simulations are shown as bars, pre-industrial (PI, 1850–1870) simulations are shown as green left-facing triangles, mid-century (MC, 1940–1960) simulations are shown as yellow circles, SSP3-7.0 scenario simulations for the end-of-century (2080–2100) period are shown as maroon right-facing triangles and SAI simulations (G6sulfur) for the end-of-century period are shown as magenta right-facing triangles. Bars and markers are coloured by the $\Delta \overline{O}$ at 27° E for each simulation. Error bars indicate the 95% confidence interval (CI) for Δ*R*, estimated from interannual variability assuming a red-noise process. Observed $\Delta \overline{R}$ and $\Delta \overline{O}$, based on the 2001–2025 EBAF record^51^ and the NSIDC snow/ice dataset^53^, are indicated by the dashed black line with grey shading (95% CI) and by a black star on the colour bar, respectively. At 27° E, ESMs fail to capture the observed E–W symmetry in $\Delta \overline{R}$ but capture the $\Delta \overline{O}$ reasonably well. **b**, $\overline{{\rm{CRE}}}$ for the EH and the WH at each model simulation’s E–W albedo symmetry (ranging from 8° to 131° E). Models are colour-coded and marker conventions follow those in **a**. Observed $\Delta \overline{{\rm{CRE}}}$ from the EBAF record^51^ over 2001–2025 (large grey circle) and its annual values (small grey dots) lie along the diagonal line denoting symmetry. All models fail to capture the triple-symmetry feature of the E–W albedo symmetry.

Present-day (1995–2015) simulations of the ESMs, as part of CMIP6’s historical experiment, diverge at the observed E–W symmetry of 27° E (bars in Fig. 4a), ranging from −2 W m^−2^ (MPI-ESM1-2) to 5.2 W m^−2^ (MIROC6). Overall, all models reproduce the observed $\Delta \overline{O}$ (colour of the bars and markers) at 27° E reasonably well, indicating a good representation of sea-ice dynamics, with the exception of MIROC6. This suggests that sea-ice coupling to the other Earth system components is not well represented. Another important result from these simulations is that, although they fail to capture the observed E–W albedo symmetry, nearly all models show a similar shift in $\Delta \overline{R}$ from pre-industrial to the ‘Regional Rivalry’ socioeconomic pathway (SSP3-7.0) at the end of century, with GISS-E2-1-H being the exception (markers in Fig. 4). Among the eight ESMs, CanESM5 exhibits the smallest $\Delta \overline{R}$ at 27° E (closest to the observed E–W symmetry), yet its end-of-century simulation under SSP3-7.0 shows the largest shift in $\Delta \overline{R}$ from its historical values. This lends support to the interpretation that Earth’s observed albedo symmetries are probably transient features of the present climate. The four G6sulfur simulations (magenta markers), representing a climate intervention scenario in which sulfate aerosols are injected into the stratosphere to scatter sunlight and cool the planet—a proposal known as stratospheric aerosol injection (SAI)—further show that large perturbations to the Earth system can alter the E–W $\Delta \overline{R}$. Within all four SAI simulations, E–W $\Delta \overline{R}$ is shifted back towards its historical values, with IPSL-CM6A-LR restoring it exactly back to its present-day value.

Another important feature that all models fail to capture is the triple-symmetry feature of the E–W albedo symmetry (Fig. 1a). To further examine this, we first identify each model’s own E–W symmetry by finding the meridian at which hemispheric $\Delta \overline{R}$ is near zero in each model’s realization. Such an analysis cannot be done in the N–S symmetry given the fixed divide at the equator. At each model’s E–W symmetry, the corresponding hemispheric difference in $\overline{{\rm{CRE}}}$ remains 5–10 W m^−2^, with GISS-E2-1-H consistently lying closer to symmetry than the other models considered (Fig. 4b). This suggests potential model deficiencies in the representation of cloud distribution that may have led to the failure to capture the observed E–W symmetry at 27° E. Also worth noting is that, although the SAI interventions shift $\Delta \overline{R}$ towards historical values, they do not act to restore $\Delta \overline{{\rm{CRE}}}$. In fact, all four SAI simulations show a pronounced reduction in hemispheric-mean CRE while maintaining a hemispheric $\Delta \overline{{\rm{CRE}}}$, relative to the SSP3-7.0 scenario (hatched versus unhatched right-facing triangles). This points to the impacts of SAI on tropospheric clouds, potentially through changes in atmospheric overturning circulation^29^ or through diffusion brightening^30^.

**Extended Data Fig. 9:**
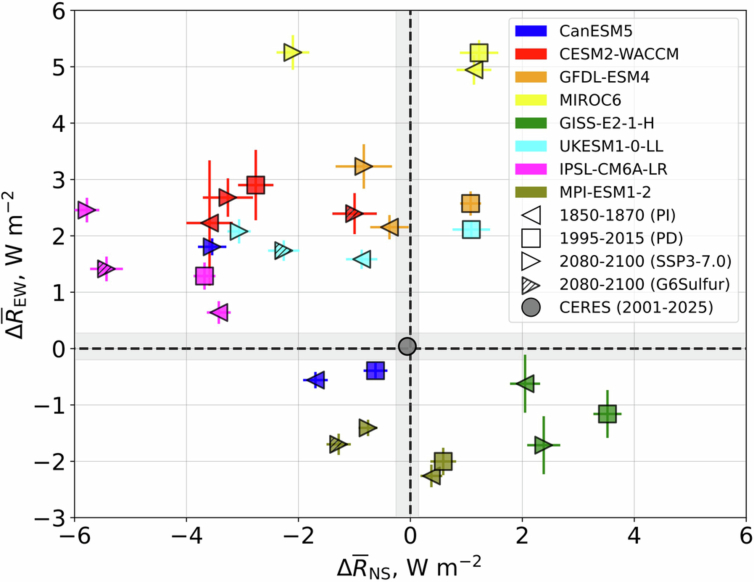
E–W and N–S asymmetries of the CMIP6 models are not clearly linked. The E–W asymmetry (at 27° E) versus the N–S asymmetry (at the equator) as captured by ESMs. Models are colour-coded, with markers denoting model experiments and error bars denoting 95% confidence. Present-day (PD, 1995–2015) simulations are shown as squares, pre-industrial (PI, 1850–1870) simulations are shown as left-facing triangles, SSP3-7.0 scenario simulations for the end-of-century (2080–2100) period are shown as right-facing triangles and SAI simulations (G6sulfur) for the end-of-century period are shown as hatched, right-facing triangles. Observed E–W and N–S symmetries (EBAF^51^, 2001–2025) are indicated by a grey circle, with shading representing 95% confidence. No clear connection between the two albedo asymmetries is found in CMIP6 model simulations.

In general, CMIP6 models show Δ*R* persistence over the examined time periods (error bars in Fig. 4a) comparable with that seen in the observed albedo symmetries (grey shading), except for GISS-E2-1-H and CESM2-WACCM^9^. Notably, CESM2-WACCM exhibits particularly large interannual variability in its pre-industrial simulation. Overall, these simulations further support that the E–W and N–S asymmetries do not appear to be directly connected (Extended Data Fig. 9). In most models, the N–S asymmetry shows larger shifts across simulated periods and scenarios than the E–W asymmetry.

## E–W symmetry tracks ENSO phase

The search for an underlying mechanism enforcing the N–S albedo symmetry has been an enduring effort but remains unresolved^5,6,8^. Recent findings, however, suggest that this search may ultimately prove futile, as the N–S symmetry has shown signs of breaking down in the recent satellite record^11^. By contrast, such an inter-hemispheric link may be more tractable in the E–W symmetry. Several lines of evidence point to the existence of such a mechanism, including the multiyear oscillation pattern in the E–W symmetry longitude (Fig. 1b) and the hemispheric compensation between low and high clouds (Fig. 2a). Together, these pieces of evidence underline the importance of an atmospheric overturning circulation—the Walker circulation^31^. Over the Pacific, the Walker circulation connects the two hemispheres (at 27° E) by coupling the Pacific warm pool with the northeastern Pacific stratocumulus deck. It has been shown that changes in subtropical low-level cloudiness over the subsiding branch of the Pacific Walker circulation can modulate tropical deep convection over its rising branch^32^, and vice versa^33^. The seasonal-to-interannual variability of the Walker circulation is associated with an oscillating climate pattern known as ENSO^31^, which alters the sea surface temperature gradient between the tropical eastern and western Pacific.

**Fig. 5:**
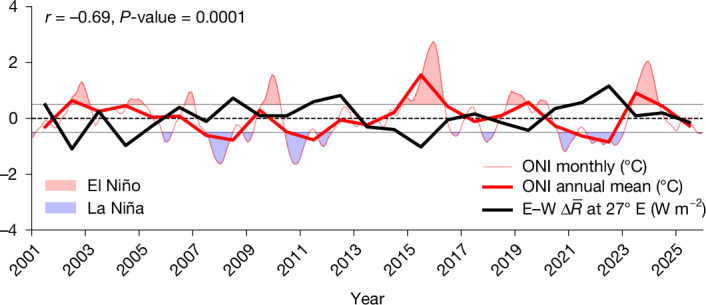
Interannual variability in the E–W symmetry tracks the phase of ENSO. Time series of annual-mean E–W hemispheric symmetry ($\Delta \overline{R}$, black) and ONI (red)^54^. Three-monthly running mean of ONI is denoted by the thin red line. Red and blue shadings indicate time periods of El Niño and La Niña events, defined as temperature anomalies exceeding 0.5 °C and −0.5 °C, respectively. The Pearson correlation coefficient between $\Delta \overline{R}$ and ONI and its *P*-value are indicated top left. A statistically significant correlation between ENSO and E–W albedo symmetry is found.

We find a strong correlation between ENSO and the interannual variability of the observed E–W hemispheric albedo symmetry at 27° E (Fig. 5). Specifically, the interannual variability of the E–W symmetry is negatively correlated with the Oceanic Niño Index (ONI), an indicator of ENSO phase (Methods), with a statistically significant correlation coefficient of −0.69 (*P*-value = 10^−4^). At the rising branches of the Walker circulation, strong convective ascent leads to expansive, bright anvil clouds capped by the tropopause, contributing substantially to TOA SW reflection. From La Niña to El Niño years, these rising branches and the corresponding subsiding branches shift zonally, reshaping cloud and precipitation patterns between E–W hemispheres. This shift in the Walker circulation could trigger cascading, potentially cross-equatorial changes in cloud distribution through coupling with meridional overturning circulations. Indeed, the most extreme interannual anomalies in the N–S symmetry have been linked to ENSO phases^9^. Our analysis of the N–S symmetry suggests a weaker and insignificant correlation between ENSO variability and the interannual variability of the N–S symmetry (*r* = −0.33, *P* = 0.11). This is consistent with the fact that ENSO is largely a mode of variability in the zonal direction. That the observed hemispheric albedo symmetries exhibit different degrees of connection to ENSO suggests that they essentially capture the ‘pulse’ of the same climate system in distinct ways. Therefore, potential alteration to atmospheric overturning circulations such as the Walker circulation under climate change^34,35^ could lead to a breakdown of Earth’s albedo symmetries.

## Discussion and implications

The connection between Earth’s planetary albedo and ENSO has important implications for how regionally or hemispherically confined perturbations can generate inter-hemispheric effects. Examples include large-scale wildfires, such as the 2019–2020 Australian bushfires^36^; volcanic eruptions, such as the 2022 Hunga-Tonga event^37^; and proposed solar radiation management^38^. In the case of marine cloud brightening^39^, climate model simulations have demonstrated that the deliberate injection of sea salt particles to enhance solar reflection from subtropical marine low clouds could affect ENSO^40,41^, with potential impacts extending to the Arctic and the stratosphere through coupling with overturning circulations^42,43^. More broadly, the discovery of the E–W albedo symmetry provides an independent line of evidence suggesting that climate intervention proposals designed to modify *R* may induce cascading impacts on regional and global cloud and precipitation patterns.

Similar to the N–S albedo symmetry, we cannot yet rule out the possibility that these hemispheric symmetries are simply coincidental features of the present climate state. In fact, accumulating evidence suggests that this may be the case^11–13^, including the findings of this work. The importance of the E–W symmetry discovery, however, is beyond the identification of another ‘sweet spot’ of the Earth system: it offers a powerful, reduced degree-of-freedom constraint on state-of-the-art ESMs and, more broadly, on our fundamental understanding of the Earth climate system. This is because its distinctive triple-symmetry feature and connection to ENSO extend the observational constraint beyond TOA all-sky reflection to the coupled behaviour of clouds, the ocean and sea ice in models. Furthermore, the inability of CMIP6 models to capture the observed characteristics of the E–W albedo symmetry suggests deficiencies in their representation of boundary-layer clouds. These deficiencies may in turn amplify or mute forcing responses and radiative feedbacks, contributing to the persistent uncertainty in climate projections^44^.

In conclusion, using more than two decades of the spaceborne Earth’s Radiation Budget record, we have discovered that the EH and the WH, separated at 27° E, reflect almost identical amounts of solar radiation. This E–W all-sky hemispheric symmetry coincides with near symmetries in ocean fraction, CRE and clear-sky reflection, together yielding a triple symmetry distinct from the N–S albedo symmetry. This unique feature provides a powerful constraint on ESMs, extending model evaluation beyond TOA reflection to the coupled representation of key Earth system components. We also find a clear connection between ENSO phases and the interannual variability of the E–W symmetry.

Amid substantial global darkening^11,25,45–47^ and a doubling of Earth’s radiation imbalance in recent decades^48^, recent satellite records suggest an emerging departure from the N–S albedo symmetry^11–13^. Although the E–W symmetry appears more robust, CMIP6 simulations across climate scenarios point to the possibility of both being transient features. Above all, our ability to test these interpretations and better understand the coupled nature of the Earth system hinges on one critical ingredient: a sustained continuation of the Earth’s Radiation Budget record^49,50^.

## Methods

### Reflected SW radiation from CERES and its decomposition

#### Products and record length

Here we obtain monthly, 1° all-sky TOA SW fluxes between January 2001 and December 2025 (inclusive) from the CERES EBAF Edition 4.2.1 product^15,16^. These data are derived from measurements of SW radiance between 0.3 and 5.0 μm from CERES instruments onboard the Terra, Aqua and NOAA-20 satellites^14–16^ and these records have been synced in the EBAF product. To decompose broadband SW reflection at TOA into components including CRE, clear-sky surface and atmospheric reflections, we obtain TOA clear-sky (for the total region) SW fluxes and calculated fluxes at the surface also from the EBAF Edition 4.2.1 product. Furthermore, to enable a cloud type decomposition of the CRE, we obtain the Flux By Cloud Type (FBCT) Edition 4A product^17^ between January 2023 and December 2022 (inclusive). There is an apparent offset in TOA SW fluxes between those measured from NOAA-20 and those measured from Terra+Aqua, preventing us from using the FBCT product beyond 2022. The FBCT product integrates information from the Moderate Resolution Imaging Spectroradiometer (MODIS) to partition CERES observed fluxes into seven cloud effective pressure (*P*_eff_) bins and six cloud optical depth (*τ*_cld_) bins. We further condense the *P*_eff_–*τ*_cld_ into nine bins, representing nine cloud types: cumulus (Cu), stratocumulus (Sc) and stratus (St) for low-level clouds capped at 680 hPa; altocumulus (Ac), altostratus (As) and nimbostratus (Ns) for mid-level clouds between 680 hPa and 440 hPa; and cirrus (Ci), cirrostratus (Cs) and cumulonimbus (Cb) for high-level clouds above 440 hPa. The separation of *τ*_cld_ for a given cloud level is set at 3.6 and 23, following ref. ^55^ and illustrated in the inset of Fig. 2a.

It worth noting that the EBAF and FBCT products are produced with different CERES data streams^15^. Specifically, in the process of producing monthly mean fluxes, the diurnal filling in the EBAF product incorporates geostationary data to capture a complete diurnal cycle^56^, whereas the FBCT product assumes a diurnally invariant cloud field fixed at the time of the CERES overpasses. This is particularly relevant here because TOA SW fluxes are known to exhibit diurnal variability owing to cloud evolution, with deep convective and stratocumulus clouds showing distinctly different diurnal cycles^57^. Moreover, the EBAF and FBCT products are also not internally synced in terms of the total TOA radiation, as EBAF is also adjusted within observational uncertainty to match observed ocean heat uptake^58^. As a result, EBAF all-sky global mean SW reflection is about 3 W m^−2^ greater than that from FBCT. Given these differences in processing streams, results may therefore appear different between the two products. In fact, the climatological E–W symmetry appears robustly at the 27° E meridian based on the EBAF record, whereas it appears robustly at the 28° E meridian based on the FBCT product. Thus, whenever the FBCT product is analysed, the 28° E meridian is used to separate the EH and the WH. The interannual variability in E–W symmetry inferred from the two products tracks closely (not shown), supporting the robustness of the results presented here.

In a nutshell, the EBAF product is used for the majority of the study for its longer record and more complete diurnal filling, and we use its latest available record from January 2001 to December 2025. The FBCT product from January 2003 to December 2022 is used when cloud type decomposition is involved.

#### Cloud radiative effect and its decomposition into cloud type

The cloud contribution to the total all-sky TOA SW reflection is defined as the CRE, calculated as the difference between all-sky reflection (*R*) and clear-sky reflection (for the total region; *R*_clr_). The CRE is then further decomposed into contributions from each cloud type, calculated as the cloud fraction (*f*_cldtyp_) weighted flux difference between upwelling overcast TOA flux for that cloud type (${F}_{{\rm{cldtyp}}}^{\uparrow }$) and the upwelling clear-sky flux (${F}_{{\rm{clr}}}^{\uparrow }$):

$${R}_{{\rm{cldtyp}}}={f}_{{\rm{cldtyp}}}({F}_{{\rm{cldtyp}}}^{\uparrow }-{F}_{{\rm{clr}}}^{\uparrow })$$

Although the CRE is not independent of clear-sky fluxes, the simplicity of its calculation offers a straightforward way to interpret cloud changes themselves.

#### Clear-sky atmospheric and surface decomposition

Clear-sky reflection *R*_clr_ is decomposed into contributions from surface (*R*_sfc_) and the atmosphere (*R*_atm_), following a simple reflection model that assumes a single-layer atmosphere^1,6,19^

$$\begin{array}{c}\begin{array}{l}{R}_{{\rm{atm}}}\,=\,S\left(\frac{{F}_{{\rm{TOA}}}^{\uparrow }{F}_{{\rm{TOA}}}^{\downarrow }-{F}_{{\rm{sfc}}}^{\uparrow }{F}_{{\rm{sfc}}}^{\downarrow }}{{({F}_{{\rm{TOA}}}^{\downarrow })}^{2}-{({F}_{{\rm{sfc}}}^{\uparrow })}^{2}}\right)\\ {R}_{{\rm{sfc}}}\,=\,S\left(\frac{{F}_{{\rm{sfc}}}^{\uparrow }}{{F}_{{\rm{TOA}}}^{\downarrow }}\right)\left(\frac{{F}_{{\rm{sfc}}}^{\downarrow }{F}_{{\rm{TOA}}}^{\downarrow }-{F}_{{\rm{sfc}}}^{\uparrow }{F}_{{\rm{TOA}}}^{\uparrow }}{{({F}_{{\rm{TOA}}}^{\downarrow })}^{2}-{({F}_{{\rm{sfc}}}^{\uparrow })}^{2}}\right)\end{array}\end{array}$$

in which *S* denotes insolation, *F* denotes SW fluxes and arrows denote the direction of the radiative flux. For the EBAF decomposition, *S* and ${F}_{{\rm{TOA}}}^{\downarrow }$ are identical. To ensure consistency with the calculated surface fluxes that are only available from EBAF, we use ${F}_{{\rm{TOA}}}^{\downarrow }$ from EBAF when calculating relative measures in the FBCT decomposition. We recognize that this decomposition is not perfect, as the surface and atmosphere components are not independent, but it remains useful, given that the overall changes in the surface component induced by atmospheric changes are small.

### Snow/ice data and surface type classification

To calculate ice-free-ocean fractions and to enable a surface type classification, we obtain monthly, 1° ‘ocean fraction coverage’ (*f*_oce_) and ‘snow/ice percent coverage’ (*f*_ice_) variables from the CERES Synoptic TOA and surface fluxes and clouds (SYN) Edition 4.2 product between 2001 and 2025 (inclusive). This product is based on the National Snow and Ice Data Center (NSIDC) daily retrievals of snow/ice coverage from the Special Sensor Microwave Imager/Sounder (SSMIS) aboard the Defense Meteorological Satellite Program (DMSP) F17 satellite overlaid on static global water and snow maps, both provided by the U.S. Geological Survey (USGS).

Using the snow/ice dataset, the Earth surface is classified into ‘ice-free land’ as regions with both *f*_ice_ < 0.5 and *f*_oce_ < 0.5; ‘ice-free ocean’ as regions with *f*_oce_ > 0.5; and ‘ice-covered’ as regions with *f*_ice_ > 0.5. This is a time-varying classification, instead of a climatological classification, such that the classification is performed at every month, using the observed snow/ice coverages at each grid. As a result, interpretation of changes in *R* associated with a given surface type under this time-varying classification should consider both changes in the areal coverage of that surface type and changes in its intrinsic reflection. This time-varying classification helps us better resolve emerging trends in *R* and its components.

### ESM simulations

We obtained simulation outputs from eight state-of-the-art coupled ESMs that participated in the CMIP6 (ref. ^26^) historical experiment, the ScenarioMIP (ref. ^27^) and the GeoMIP6 (ref. ^28^). The models were selected to maximize the representation of variabilities in both model architecture and climate scenarios. To do this, we first grouped CMIP6 models into 14 families based on Fig. 2 in ref. ^59^. We then selected eight of out the 14 families that have an ESM, as ocean coupling is a critical part of Earth albedo symmetries. The IITM-ESM family was excluded because of the lack of the sea ice (‘siconc’) variable. Within each of the eight families, we then selected models that participated in both historical and SSP3-7.0 experiments, with a preference given to the model that also participated in the G6sulfur experiment (as part of GeoMIP). This selection process yields a total of eight models, one from each family: NOAA Geophysical Fluid Dynamics Laboratories GFDL-ESM4 (GFDL, variant: r1i1p1f1)^60,61^, NASA Goddard Institute for Space Studies GISS-E2-1-H (GISS, variant: r1i1p1f2)^62,63^, Institut Pierre-Simon Laplace IPSL-CM6A-LR (IPSL, variant: r1i1p1f1)^64–66^, University of Tokyo, National Institute for Environmental Studies and Japan Agency for Marine-Earth Science and Technology MIROC6 (MIROC, variant: r1i1p1f1)^67,68^, the UK Met Office Hadley Centre Natural Environment Research Council UKESM1-0-LL (UKESM, variant: r1i1l1f2)^69,70^, Canadian ESM version 5 (CanESM5, variant: r10i1p1f1)^71,72^, National Center of Atmospheric Research CESM2-WACCM (CESM2, variant: r1i1p1f1 and r1i1p1f1 for G6sulfur)^73–75^ and Max-Planck Institute MPI-ESM1-2 (MPI, variant: r1i1p1f1)^76–78^. Only one variant is obtained from each model for analysis. We obtain ‘rsut’ (TOA all-sky SW reflection), ‘rsutcs’ (TOA all-sky SW reflection assuming clear-sky) and ‘siconc’ (sea ice area fraction) variables from these model simulations. TOA flux decomposition is calculated in the same way as in the CERES products (CRE = rsut − rsutcs).

### Oceanic Niño Index

To indicate the phase of ENSO, we obtain the ONI from 2001 to 2025 (inclusive), the primary indicator for monitoring the oceanic part of ENSO, from the Climate Prediction Center at the National Oceanic and Atmospheric Administration (NOAA). The ONI is calculated as the 3-month running average sea surface temperature anomalies in the east-central tropical Pacific between 120° and 170° W (known as the Niño3.4 region) relative to the 1981–2010 average. ONI values of +0.5 °C or higher (−0.5 °C or lower) are considered El Niño (La Niña) conditions by the NOAA.

### Spatiotemporally weighted averaging, statistical significance and trend analysis

In calculations of hemispheric, regional, zonal and global climatologies, spatially and temporally weighted averaging is performed, taking into account the difference in grid box size, month length and the geometry of the Earth as an oblate spheroid. This spatiotemporally weighted averaging is performed on the basis of each climate model’s specific grid configurations for oceanic and atmospheric variables, using the static ‘grid-cell area’ variable.

Uncertainty in the climatological E–W symmetry from interannual variability is quantified assuming a red noise process, based on the lag-1 autocorrelation^79^. Statistical significance is quantified by a standard Student’s *t*-test at the 95% confidence interval. We calculate trends from time series of deseasonalized anomalies independently at each grid and later aggregated to regional, zonal, hemispheric and global averages.

## Online content

Any methods, additional references, Nature Portfolio reporting summaries, source data, extended data, supplementary information, acknowledgements, peer review information; details of author contributions and competing interests; and statements of data and code availability are available at 10.1038/s41586-026-10624-2.

## Supplementary information

Peer Review File

## Data Availability

The CERES products (FBCT and EBAF) used in this study were obtained from the NASA’s Langley Research Center CERES ordering tool at https://ceres.larc.nasa.gov/data/ (downloaded 15 April 2026) and are available at 10.5067/Terra-Aqua/CERES/FLUXBYCLDTYP-MONTH_L3.004A (ref. ^52^) and 10.5067/TERRA-AQUA-NOAA20/CERES/EBAF_L3B004.2.1 (ref. ^51^). The NSIDC snow/ice coverage data were obtained through the CERES Synoptic TOA and surface fluxes and clouds (SYN) Edition 4.2 product (downloaded 1 March 2026) and are available at 10.5067/TERRA+AQUA/CERES/SYN1DEGMONTH_L3.004A (ref. ^53^). CMIP6, ScenarioMIP and GeoMIP6 data are available from the Earth System Grid Federation (ESGF) and were downloaded from the US Department of Energy/Lawrence Livermore National Laboratory node at https://esgf-node.llnl.gov/projects/cmip6/ (downloaded 1 March 2026). References to individual ESMs and their experiments are summarized in Methods. The ONI data were obtained from the Climate Prediction Center at NOAA (downloaded 1 March 2026) and are publicly available at https://www.cpc.ncep.noaa.gov/data/indices/oni.ascii.txt (ref. ^54^). Maps with coastlines were produced using Cartopy^80^ in the Orthographic and PlateCarree projections.

## Code availability

All analytical and plotting scripts required to reproduce the figures and results presented in this paper are publicly available at https://csl.noaa.gov/groups/csl9/datasets/data/2026-Zhang/ (ref. ^81^).

## References

[1] Stephens, G. L. et al. The albedo of Earth. *Rev. Geophys.* **53**, 141–163 (2015).

[2] Vonder Haar, T. H. & Suomi, V. E. Measurements of the earth’s radiation budget from satellites during a five-year period. Part I: extended time and space means. *J. Atmos. Sci.* **28**, 305–314 (1971).

[3] Ramanathan, V. The role of earth radiation budget studies in climate and general circulation research. *J. Geophys. Res. Atmos.* **92**, 4075–4095 (1987).

[4] Stevens, B. & Schwartz, S. E. Observing and modeling Earth’s energy flows. *Surv. Geophys.* **33**, 779–816 (2012).

[5] Bender, F. A.-M., Engström, A., Wood, R. & Charlson, R. J. Evaluation of hemispheric asymmetries in marine cloud radiative properties. *J. Clim.* **30**, 4131–4147 (2017).

[6] Diamond, M. S., Gristey, J. J. & Feingold, G. Testing cloud adjustment hypotheses for the maintenance of Earth’s hemispheric albedo symmetry with natural experiments. *Geophys. Res. Lett.* **51**, e2024GL111733 (2024).

[7] Voigt, A., Stevens, B., Bader, J. & Mauritsen, T. The observed hemispheric symmetry in reflected shortwave irradiance. *J. Clim.* **26**, 468–477 (2013).

[8] Datseris, G. & Stevens, B. Earth’s albedo and its symmetry. *AGU Adv.* **2**, e2021AV000440 (2021).

[9] Jönsson, A. & Bender, F. A.-M. Persistence and variability of Earth’s interhemispheric albedo symmetry in 19 years of CERES EBAF observations. *J. Clim.* **35**, 249–268 (2022).

[10] Diamond, M. S., Gristey, J. J., Kay, J. E. & Feingold, G. Anthropogenic aerosol and cryosphere changes drive Earth’s strong but transient clear-sky hemispheric albedo asymmetry. *Commun. Earth Environ.* **3**, 206 (2022). 36118252 10.1038/s43247-022-00546-y PMC9466336

[11] Loeb, N. G. et al. Emerging hemispheric asymmetry of Earth’s radiation. *Proc. Natl Acad. Sci. USA* **122**, e2511595122 (2025). 41021814 10.1073/pnas.2511595122 PMC12519167

[12] Oreopoulos, L., Cho, N. & Lee, D. The role of Earth’s major cloud systems in the hemispheric albedo symmetry. *J. Clim.* **38**, 7203–7215 (2025).

[13] Singer, C. E. & Pincus, R. Southern Ocean clear-sky brightening from sea spray aerosol increase drives departure from hemispheric albedo symmetry. *Geophys. Res. Lett.* **53**, e2025GL119637 (2025).

[14] Wielicki, B. A. et al. Clouds and the Earth’s Radiant Energy System (CERES): an Earth observing system experiment. *Bull. Am. Meteorol. Soc.* **77**, 853–868 (1996).

[15] Loeb, N. G. et al. Clouds and the Earth’s Radiant Energy System (CERES) Energy Balanced and Filled (EBAF) Top-of-Atmosphere (TOA) Edition-4.0 data product. *J. Clim.* **31**, 895–918 (2018).

[16] Kato, S. et al. Surface irradiances of Edition 4.0 Clouds and the Earth’s Radiant Energy System (CERES) Energy Balanced and Filled (EBAF) data product. *J. Clim.* **31**, 4501–4527 (2018).

[17] Sun, M. et al. Clouds and the Earth’s Radiant Energy System (CERES) FluxByCldTyp Edition 4 data product. *J. Atmos. Ocean. Technol.* **39**, 303–318 (2022).

[18] Klein, S. A. & Hartmann, D. L. The seasonal cycle of low stratiform clouds. *J. Clim.* **6**, 1587–1606 (1993).

[19] Donohoe, A. & Battisti, D. S. Atmospheric and surface contributions to planetary albedo. *J. Clim.* **24**, 4402–4418 (2011).

[20] Cesana, G. V. et al. The correlation between Arctic sea ice, cloud phase and radiation using A-Train satellites. *Atmos. Chem. Phys.* **24**, 7899–7909 (2024).

[21] Cavalieri, D. J. & Parkinson, C. L. Arctic sea ice variability and trends, 1979–2010. *Cryosphere* **6**, 881–889 (2012).

[22] Eayrs, C., Li, X., Raphael, M. N. & Holland, D. M. Rapid decline in Antarctic sea ice in recent years hints at future change. *Nat. Geosci.* **14**, 460–464 (2021).

[23] Chen, C. et al. China and India lead in greening of the world through land-use management. *Nat. Sustain.* **2**, 122–129 (2019). 30778399 10.1038/s41893-019-0220-7 PMC6376198

[24] Davies, T. W. & Smyth, T. Darkening of the global ocean. *Glob Change Biol.* **31**, e70227 (2025). 10.1111/gcb.70227 PMC12107402 40421554

[25] Tselioudis, G., Remillard, J., Jakob, C. & Rossow, W. B. Contraction of the world’s storm-cloud zones the primary contributor to the 21st century increase in the Earth’s sunlight absorption. *Geophys. Res. Lett.* **52**, e2025GL114882 (2025).

[26] Eyring, V. et al. Overview of the Coupled Model Intercomparison Project Phase 6 (CMIP6) experimental design and organization. *Geosci. Model Dev.* **9**, 1937–1958 (2016).

[27] O’Neill, B. C. et al. The Scenario Model Intercomparison Project (ScenarioMIP) for CMIP6. *Geosci. Model Dev.* **9**, 3461–3482 (2016).

[28] Kravitz, B. et al. The Geoengineering Model Intercomparison Project Phase 6 (GeoMIP6): simulation design and preliminary results. *Geosci. Model Dev.* **8**, 3379–3392 (2015).

[29] McGraw, Z. & Polvani, L. M. Direct radiative impacts of stratospheric aerosols on the tropical troposphere: clouds, precipitation, and circulation in convection-resolving and global simulations. Preprint at https://arxiv.org/abs/2512.06163 (2025).

[30] Gristey, J. J. & Feingold, G. Stratospheric aerosol injection would change cloud brightness. *Geophys. Res. Lett.* **52**, e2024GL113914 (2025).

[31] Bjerknes, J. Atmospheric teleconnections from the equatorial Pacific. *Mon. Weather Rev.* **97**, 163–172 (1969).

[32] Dagan, G., Yeheskel, N. & Williams, A. I. L. Radiative forcing from aerosol-cloud interactions enhanced by large-scale circulation adjustments. *Nat. Geosci.* **16**, 1092–1098 (2023).

[33] McCulloch, D., Webb, M. J., Lambert, F. H. & Vallis, G. K. Weakening tropical deep convection reduces subtropical low clouds via lower free-tropospheric moisture convergence in a climate model. Preprint at https://www.authorea.com/doi/full/10.22541/au.175407740.01003894 (2025).

[34] Held, I. M. & Soden, B. J. Robust responses of the hydrological cycle to global warming. *J. Clim.* **19**, 5686–5699 (2006).

[35] Vecchi, G. A. & Soden, B. J. Global warming and the weakening of the tropical circulation. *J. Clim.* **20**, 4316–4340 (2007).

[36] Fasullo, J. T., Rosenbloom, N. & Buchholz, R. A multiyear tropical Pacific cooling response to recent Australian wildfires in CESM2. *Sci. Adv.* **9**, eadg1213 (2023). 37163592 10.1126/sciadv.adg1213 PMC10171808

[37] Zhu, Y., Mann, G., Newman, P. A. & Randel, W. The Hunga volcanic eruption atmospheric impacts report. APARC IPO, APARC report 11, 10.34734/FZJ-2025-05237 (2025).

[38] National Academies of Sciences, Engineering, and Medicine. *Reflecting Sunlight: Recommendations for Solar Geoengineering Research and Research Governance* (The National Academies Press, 2021).

[39] Feingold, G. et al. Physical science research needed to evaluate the viability and risks of marine cloud brightening. *Sci. Adv.* **10**, eadi8594 (2024). 38507486 10.1126/sciadv.adi8594 PMC10954212

[40] Hill, S. & Ming, Y. Nonlinear climate response to regional brightening of tropical marine stratocumulus. *Geophys. Res. Lett.* **39**, L15707 (2012).

[41] Xing, C. Subtropical marine cloud brightening suppresses the El Niño–Southern Oscillation. *Earths Future* **13**, e2025EF006522 (2025).

[42] Rasch, P. J., Latham, J. & Chen, C.-C. J. Geoengineering by cloud seeding: influence on sea ice and climate system. *Environ. Res. Lett.* **4**, 045112 (2009).

[43] Bednarz, E. M., Haywood, J. M., Visioni, D., Butler, A. H. & Jones, A. How marine cloud brightening could also affect stratospheric ozone. *Sci. Adv.* **11**, eadu4038 (2025). 40367162 10.1126/sciadv.adu4038 PMC12077500

[44] Forster, P. et al. in *Climate Change 2021: The Physical Science Basis. Contribution of Working Group I to the Sixth Assessment Report of the Intergovernmental Panel on Climate Change* (eds Masson-Delmotte, V. et al.) 923–1054 (Cambridge Univ. Press, 2021).

[45] Loeb, N. G. et al. Satellite and ocean data reveal marked increase in Earth’s heating rate. *Geophys. Res. Lett.* **48**, e2021GL093047 (2021).

[46] Kramer, R. J. et al. Observational evidence of increasing global radiative forcing. Geophys. Res. Lett. 48, e2020GL091585 (2021).

[47] Loeb, N. G. et al. Observational assessment of changes in Earth’s energy imbalance since 2000. *Surv. Geophys.* **45**, 1757–1783 (2024). 39734427 10.1007/s10712-024-09838-8 PMC11671437

[48] Mauritsen, T. et al. Earth’s energy imbalance more than doubled in recent decades. *AGU Adv.* **6**, e2024AV001636 (2025).

[49] Loeb, N. G. et al. Continuity in top-of-atmosphere Earth radiation budget observations. *J. Clim.* **37**, 6093–6108 (2024).

[50] KISS Continuity Study Team. Toward a US framework for continuity of satellite observations of Earth’s climate and for supporting societal resilience. *Earths Future* **12**, e2023EF003757 (2024).

[51] NASA/LARC/SD/ASDC. CERES Energy Balanced and Filled (EBAF) TOA and Surface Monthly means data in netCDF Edition 4.2.1. 10.5067/TERRA-AQUA-NOAA20/CERES/EBAF_L3B004.2.1 (2025).

[52] NASA/LARC/SD/ASDC. CERES Monthly Daytime Mean Regionally Averaged Terra and Aqua TOA Fluxes and Associated Cloud Properties Stratified by Optical Depth and Effective Pressure Edition4A. 10.5067/Terra-Aqua/CERES/FLUXBYCLDTYP-MONTH_L3.004A (2020).

[53] NASA/LARC/SD/ASDC. CERES and GEO-Enhanced TOA, Within-Atmosphere and Surface Fluxes, Clouds and Aerosols Monthly Terra-Aqua Edition4A. 10.5067/TERRA+AQUA/CERES/SYN1DEGMONTH_L3.004A (2017).

[54] NOAA Climate Prediction Center. Oceanic Niño Index (ONI). https://www.cpc.ncep.noaa.gov/data/indices/oni.ascii.txt (2026).

[55] Rossow, W. B. & Schiffer, R. A. Advances in understanding clouds from ISCCP. *Bull. Am. Meteorol. Soc.* **80**, 2261–2287 (1999).

[56] Doelling, D. R. et al. Geostationary enhanced temporal interpolation for CERES flux products. *J. Atmos. Ocean. Technol.* **30**, 1072–1090 (2013).

[57] Gristey, J. J. et al. Insights into the diurnal cycle of global Earth outgoing radiation using a numerical weather prediction model. *Atmos. Chem. Phys.* **18**, 5129–5145 (2018).

[58] Loeb, N. G. et al. Toward optimal closure of the Earth’s top-of-atmosphere radiation budget. *J. Clim.* **22**, 748–766 (2009).

[59] Kuma, P., Bender, F. A. & Jönsson, A. R. Climate model code genealogy and its relation to climate feedbacks and sensitivity. *J. Adv. Model. Earth Syst.* **15**, e2022MS003588 (2023).

[60] Krasting, J. P. et al. NOAA-GFDL GFDL-ESM4 model output prepared for CMIP6 CMIP historical. Earth System Grid Federation. 10.22033/ESGF/CMIP6.859 (2018).

[61] Guo, H. et al. NOAA-GFDL GFDL-CM4 model output prepared for CMIP6 ScenarioMIP. Earth System Grid Federation. 10.22033/ESGF/CMIP6.9242 (2018).

[62] NASA Goddard Institute For Space Studies (NASA/GISS). NASA-GISS GISS-E2.1G model output prepared for CMIP6 CMIP historical. Earth System Grid Federation. 10.22033/ESGF/CMIP6.7127 (2018).

[63] NASA Goddard Institute For Space Studies (NASA/GISS). NASA-GISS GISS-E2.1G model output prepared for CMIP6 ScenarioMIP. Earth System Grid Federation. 10.22033/ESGF/CMIP6.2074 (2020).

[64] Boucher, O. et al. IPSL IPSL-CM5A2-INCA model output prepared for CMIP6 CMIP historical. Earth System Grid Federation. 10.22033/ESGF/CMIP6.13661 (2020).

[65] Boucher, O. et al. IPSL IPSL-CM5A2-INCA model output prepared for CMIP6 ScenarioMIP. Earth System Grid Federation. 10.22033/ESGF/CMIP6.15667 (2020).

[66] Boucher, O. et al. IPSL IPSL-CM6A-LR model output prepared for CMIP6 GeoMIP G6sulfur. Earth System Grid Federation. 10.22033/esgf/cmip6.5059 (2020).

[67] Tatebe, H. & Watanabe, M. MIROC MIROC6 model output prepared for CMIP6 CMIP historical. Earth System Grid Federation. 10.22033/ESGF/CMIP6.5603 (2018).

[68] Shiogama, H., Abe, M. & Tatebe, H. MIROC MIROC6 model output prepared for CMIP6 ScenarioMIP. Earth System Grid Federation. 10.22033/ESGF/CMIP6.898 (2019).

[69] Tang, Y. et al. MOHC UKESM1.0-LL model output prepared for CMIP6 CMIP historical. Earth System Grid Federation. 10.22033/ESGF/CMIP6.6113 (2019).

[70] Good, P. et al. MOHC UKESM1.0-LL model output prepared for CMIP6 ScenarioMIP. Earth System Grid Federation. 10.22033/ESGF/CMIP6.1567 (2019).

[71] Swart, N. C. et al. CCCma CanESM5 model output prepared for CMIP6 CMIP historical. Earth System Grid Federation. 10.22033/ESGF/CMIP6.3610 (2019).

[72] Swart, N. C. et al. CCCma CanESM5 model output prepared for CMIP6 ScenarioMIP. Earth System Grid Federation. 10.22033/ESGF/CMIP6.1317 (2019).

[73] Danabasoglu, G. NCAR CESM2 model output prepared for CMIP6 CMIP historical. Earth System Grid Federation. 10.22033/ESGF/CMIP6.7627 (2019).

[74] Danabasoglu, G. NCAR CESM2 model output prepared for CMIP6 ScenarioMIP. Earth System Grid Federation. 10.22033/ESGF/CMIP6.2201 (2019).

[75] Danabasoglu, G. NCAR CESM2-WACCM model output prepared for CMIP6 GeoMIP. Earth System Grid Federation. 10.22033/ESGF/CMIP6.10025 (2019).

[76] Wieners, K.-H. et al. MPI-M MPI-ESM1.2-LR model output prepared for CMIP6 CMIP historical. Earth System Grid Federation. 10.22033/ESGF/CMIP6.6595 (2019).

[77] Wieners, K.-H. et al. MPI-M MPIESM1.2-LR model output prepared for CMIP6 ScenarioMIP. Earth System Grid Federation. 10.22033/ESGF/CMIP6.793 (2019).

[78] Niemeier, U. et al. MPI-M MPI-ESM1.2-LR model output prepared for CMIP6 GeoMIP G6sulfur. Earth System Grid. 10.22033/ESGF/CMIP6.6448 (2019).

[79] Bretherton, C. S., Widmann, M., Dymnikov, V. P., Wallace, J. M. & Bladé, I. The effective number of spatial degrees of freedom of a time-varying field. *J. Clim.* **12**, 1990–2009 (1999).

[80] Met Office. Cartopy: a cartographic Python library with a Matplotlib interface. https://cartopy.readthedocs.io (2010–2015).

[81] Zhang, J. Analytical and plotting scripts for “Earth’s east–west albedo symmetry”. https://csl.noaa.gov/groups/csl9/datasets/data/2026-Zhang/ (2026). 10.1038/s41586-026-10624-2 PMC13275288 42236934

[82] Xu, R. et al. Contrasting impacts of forests on cloud cover based on satellite observations. *Nat. Commun.* **13**, 670 (2022). 35115519 10.1038/s41467-022-28161-7 PMC8813950

[83] Leung, G. R., Grant, L. D. & van den Heever, S. C. Deforestation-driven increases in shallow clouds are greatest in drier, low-aerosol regions of Southeast Asia. *Geophys. Res. Lett.* **51**, e2023GL107678 (2024).

[84] Dror, T. & Feingold, G. Amazon forest loss: an all-sky biophysical top-of-atmosphere cooling feedback. *Science* **392**, 429–432 (2026). 42024762 10.1126/science.adz8296

[85] Sévellec, F., Fedorov, A. V. & Liu, W. Arctic sea-ice decline weakens the Atlantic Meridional Overturning Circulation. *Nat. Clim. Change* **7**, 604–610 (2017).

[86] Liu, W., Fedorov, A. & Sévellec, F. The mechanisms of the Atlantic meridional overturning circulation slowdown induced by Arctic sea ice decline. *J. Clim.* **32**, 977–996 (2019).

[87] Weijer, W. Interactions between the Arctic Mediterranean and the Atlantic Meridional Overturning Circulation: a review. *Oceanography* **35**, 118–127 (2022).

