# Supplementary material for: Earth’s east–west albedo symmetry
Source: Nature. 2026 Jun 3;654(8119):676–82. doi: 10.1038/s41586-026-10624-2 (PMC13275288; doi:10.1038/s41586-026-10624-2)
Supplement: Supplementary file 1 — Peer Review File [file 41586_2026_10624_MOESM1_ESM.pdf]

nature portfolio

Peer Review File

Earth's East-West albedo symmetry

Corresponding Author: Dr Jianhao Zhang

Any redactions in this file are there to maintain patient confidentiality, the confidentiality of unpublished data, or to remove third-party material.

This file contains all reviewer reports in order by version, followed by all author rebuttals in order by version.

Attachments originally included by the reviewers as part of their assessment can be found at the end of this file.

Version 0:

Reviewer comments:

Referee #1

(Remarks to the Author)
Review of a paper by Zhang et al. entitled “Earth’s albedo symmetry at 28 degree E” submitted to Nature.

The authors found that the 28-degree east meridian divides eastern and western hemisphere with an equal annual mean open water fraction. In addition, the eastern and western hemisphere have the same clear-sky and cloudy sky albedos. This implies that cloud contributions to the albedo for the eastern and western hemispheres are the same. Because the western hemisphere contains marine stratocumulus regions, this also implies that high clouds in the eastern hemisphere compensate a smaller contribution from low level clouds in the eastern hemisphere. Furthermore, in the last 20 years, the western hemisphere is darkened due to decreased cloud amount while the eastern hemisphere is darkened due to decreased land albedo. The authors also found a correlation between albedos of cryosphere and lower-latitude land regions. The authors investigated whether climate models can produce the eastern-western hemispherical albedo symmetry. While most of the tested models fail to produce the symmetry, the bias of the symmetry seems to be caused by the bias of maintaining the symmetry of open water area. For the cloud contribution to the symmetry, the authors found that only the GISS model maintains the cloud contribution symmetry.

The eastern-western albedo symmetry that the authors discovered is interesting. Also, the manuscript is well written and discusses authors’ thoughts on the symmetry. However, the manuscript does not explain beyond this curious nature of the symmetry. The community accepted that the northern hemisphere and southern hemisphere albedo symmetry is a property of the current climate system. Whether this property is the intrinsic nature of the climate system and holds for the future climate are debatable. Given that, the manuscript provides an open-ended question of whether the albedo symmetry is one of intrinsic properties of the climate system. What is missing in this manuscript is, however, to link the eastern-western hemispheric albedo symmetry to the northern-southern hemispheric albedo symmetry. It is possible that two albedo symmetries are caused by completely different reasons, but the authors can apply similar analyses to both symmetries. For example, performing a similar analysis shown in Figure 2 to northern-southern hemispherical hemispheres might give us a clue of fundamental requirements for the symmetry.
I understand that the eastern-western hemispheric albedo symmetry is different from northern-southern hemispheric albedo symmetry because the former has the same clear-sky albedo while the northern hemisphere has a larger clear-sky albedo. However, clouds compensate darkening land in the former symmetry might provide a clue to the latter. I believe that the linking these two albedo symmetries is important for this manuscript so that it makes this finding transferring from a merely curious finding to investigating intrinsic properties of the climate system. In doing so, I suggest the authors to come up with a working hypothesis for requirements of the albedo symmetry, justifying the motivation of finding eastern-western hemispherical albedo symmetry (probably right before line 22), and testing the hypothesis. Although some of authors’ thoughts are given in the current version, making a hypothesis and justifying finding of the eastern-western hemispheric albedo symmetry makes the manuscript flow better. If, however, the authors argue that the eastern-western hemispheric albedo symmetry is not related to northern-southern hemispheric albedo symmetry, please justify the scientific motivation of finding the symmetry and describe conclusions accordingly.

Minor comments

Figure 1: the waterfall chart is confusing. Could you explain what the gray bars represent? Also, it is not clear how values on the y-axis are related to other color bars. If the y values are not related with colored bars, I suggest using a simple bar chart.

Line-138-140: Because the fitted magenta line goes through the origin in Figure 3a, does this imply that if a climate model were to have the correct open ocean area symmetry, it would have modeled the albedo symmetry correctly? In other words, does the failure of modeling albedo symmetry come from the failure of modeling sea ice symmetry?

Figure 3: I am puzzled by the differences of the x-axis values of data points between figure 2a and 2b. If Figure 2b replaces the y-axis of Figure 2a by longitude, I expect that all points in Figure 2b have the same x values with Figure 2a.

Methods: When computing hemispherical means, are 1 degree by 1 degree areas weighted by the area?

Referee #2

(Remarks to the Author)
The fact that the Northern and Southern Hemispheres reflect approximately equal amounts of solar radiation has been a widely documented and studied topic, although no definitive explanation has so far been given as to why this state of affairs should exist. Actually, two of the three authors of this study have co-authored a paper that examined aspects of this NH-SH symmetry, namely in what this manuscript labels as citation [9], wherein some figures, like the waterfall plots, look exactly like the ones here. In this study, the authors present on the discovery of a previously unknown symmetry in the way the planet reflects sunlight. Two hemispheres are again involved, but this time the demarcation line is not the equator, but the 28°E meridian, in a climatological sense: in short, instead of northern and southern hemispheres, now we are dealing with eastern and western hemispheres (EH and WH) reflecting approximately equal amounts of sunlight. Contrary to the N-S split for which the symmetry makes sense at temporal scales of a year or multiples thereof (with some adjustments to account for subtle differences between leap and non-leap years, see Material and Methods in citation [10]), because of the seasonal dependence the insolation that the N-S hemispheres receive, the E-W split can in principle be examined at scales as short as a day and multiples thereof from an insolation symmetry perspective. Curiously, this important distinction is not mentioned in the manuscript. While Fig. 1c in [9] didn't make sense to me, the same figure (reoriented) in this manuscript, also Fig. 1c, now makes total sense. Along the same lines, extended Fig. 5 does not make sense for the N-S symmetry, but is entirely legitimate for the E-W symmetry. It is important to emphasize a point that may be missed by a superficial examination of the paper: the 28°E meridian is the median of longitudes where symmetry of reflected fluxes between EH and WH occurs as monthly values of reflectance are being accumulated; for individual years (and presumably shorter time scales) a different meridian defines the equally reflecting hemispheres (Fig. 1b).

So is this (well-written and organized) paper worthy of publication in Nature, provided some issues are addressed? It is a tough call. I am leaning no for Nature proper specifically, but open to change my mind, and am certainly positive to publications of lesser stature. A compelling new (as far as I know) finding is documented here about which the authors appear to be genuinely excited; but like the NH-SH symmetry, it remains more of a curiosity without a plausible physical link to the workings of the Earth System. It should be noted that while there may be no explanation for why the mean state of the Earth has symmetric reflectance around 28° (so it could be fortuitous), it seems that deviations from symmetry (more on that later) are linked to ENSO which affects the Walker circulation which in turn affects the meridional distribution of cloudiness. This is one of the important findings of the paper (Fig. 4) that could serve as springboard for further studies.

I have both major and minor points on certain aspects of the analysis and the presentation of the results. Not sure whether the major ones will trigger revisions in the calculations, but I want to help ensure that something didn't go amiss in the calculations.

Major points
1. I think it is critical that the interannual variability and trends are discussed with maximum possible clarity. The 28°E meridian is a median longitude (L47) of different longitudes defining EH and WH with equal solar reflected fluxes, right? First of all why are you defining this "golden" meridian this way? Why not find the meridian that makes the 20-year climatological reflected fluxes of two hemispheres equal? In any case, the bottom line is that for individual years equality is achieved at different longitudes (Fig. 1b). In other words, for individual years there is in general no symmetry at 28°E. So what is then the meaning of the trend in the (a)symmetry in figures such as Fig. 1c? Is 28°E fixed and a linear fit is applied to those deviations from symmetry (L95-96)? You can always find symmetry for individual years as long as you move the meridian, so the trend is then zero for a moving meridian. But I guess then that the trend shown in the thick black line of Fig 2c comes from a linear fit to the blue line of Fig. 4?
2. I don't understand R=Rclr+Rcld at any spatial scale/domain and either of the two CERES products, FBCT or EBAF. If R is the all-sky TOA flux, shouldn't it be R=(1-CF)Rclr+CF*Rcld which is consistent with the definition of CRE=R-Rclr=CF(Rcld-Rclr)? There has to be weighting in the summation, unless the clr and cld fluxes have already been pre-weighted which is not stated anywhere as far as I can tell. See eq. (6)-(8) in citation [6]. While FBCT gives you Rcld, and also its breakdown by cloud type, EBAF gives only all-sky R and clear-sky Rclr. But you can certainly not get the gridbox flux in FBCT by just summing Rclr and Rcld; you have to weight by the area of the clear and cloudy portions. By the same token you can't get Rcld as simply the sum of cloud fluxes of cloud types (L224), you have to take weighted average by cloud type population. I'm not sure whether this simply a matter of these relationships being shown symbolically rather than explicitly and rigorously or the calculations being done incorrectly. This is certainly merits clarification, especially when other types of weighted averaging (like accounting for different number of days in a month) are documented.
3. How do you get a gridcell's Rcld, which I interpret as overcast cloud flux, from a climate model? Again, to my knowledge,

a model gives you only R and Rclr. Unless you obtain it through the model's vertically projected planar cloud fraction, CF. But CF is not a variable that appears anywhere in the manuscript, in contrast to [6].
4. Why is only Rclr decomposed to surface and atmosphere contributions? In cloudy columns, Rcld does not come only from the cloud alone (the terminology is not exact throughout the paper, it's the reflectance of the cloudy column rather than the reflectance of the cloud), but from the combined contributions of cloud, surface, and (cloudless) atmosphere. When the cloud is of small or moderate opacity over a land or ice surface (two situations explicitly resolved), the surface contribution can be substantial. The decomposition of reflected flux as performed in the paper is therefore incomplete in my opinion. If nothing is done about it, this should at minimum be identified and discussed.
5. Isn't the enthusiasm about the skill of the GISS model in reproducing the E-W symmetry tempered by the fact that the absolute values of reflected flux are so biased? PD values of around 103 Wm-2 (extended Fig. 8c), corresponding to a planetary albedo of 0.30, compared to ~96 Wm-2 in observations (Fig. 1a,b, presumably FBCT), corresponding to a planetary albedo of 0.28, and ~99 Wm-2 from EBAF (e.g., Oreopoulos et al. 2025), corresponding to a planetary albedo of 0.29 (the value quoted in the paper)? 4 and 7 Wm-2 are huge biases! I would probably trust more a climate model that gives me correct current state planetary albedo than one that gives me the most symmetry around 28°E.
6. Have the authors considered examining (a)symmetry around 28E of other energy components besides reflected flux? OLR, the atmosphere's radiative cooling? How close or far are the two EH or WH in radiative convective equilibrium (if this something that matters in explaining the symmetry phenomenon)? Other energy components are examined in N-S symmetry papers, for example in Oreopoulos et al. (2025) and citation [10].
7. In the lag analysis, are the authors basically implying that there are processes involving clouds that operate on the scale of a full year?

Miscellaneous points of wide-ranging importance
1. Abstract: Why is the albedo symmetry "delicate"? In what sense? "Each of which ALSO persistently". Why "also"? What else do these hemispheres do? You haven't said yet that they have equal land-ocean ratios. "reflectS" instead of "reflect". "at this meridian" or "around this meridian"?
2. Lines 17-21: 3% is still more than what you find here (n=1), no? My understanding is that the areas making the equal hemispheres in the cited study were allowed to be non-contiguous. So your finding is much more special!
3. L35-36: "symmetry in cloud reflection". See my major point (4). Cloud reflectance may not be symmetric, but the contributions from everything else in the cloudy column make it symmetric. See also L85-86.
4. L95-96: It's not the trend in R, but rather ΔR, right?
5. I'm not a big fan of Fig. 1a. Longitude as color, what a strange choice, but I've convinced myself to accept the intent to show that flux asymmetry and ocean fraction symmetry have a monotonic relationship which is a recurring theme in the paper. But Fig. 1b? Why keep showing ΔO? Why not show longitude of ΔR=0 vs year?
6. Figure 3 is also curious. ΔR and longitude are never shown on the same plot, as in "what is the longitude where ΔR=0?". It's just a convoluted way of showing things with the choices made for Fig. 3.
7. Why are the authors showing the sum of the hemispheric values (the numbers in the neighborhood of 200 Wm-2) in extended Figures 5 and 8?
8. L210, to be exact these are equatorial daytime crossing times.
9. L218, please cite the primary ISCCP literature for 3.6 and 23 and not Diamond et al. which is a secondary reference.
10. In the description of CERES data, I am sure the authors are aware that by going up to December 2024 in EBAF, NOAA-20 satellite data are also involved.
11. In the equations used to derive Ratm and Rsfc isn't "S" and FTOA_down the same thing?
12. Why were only the particular 5 climate models selected? Were these the only ones that had performed all the experiments the authors wanted to include?

Referee #3

(Remarks to the Author)
Review of: "Earth's albedo symmetry at 28° E", Zhang et al.

The authors report an interesting east-west hemispheric symmetry of albedo contributions by the surface, clouds, and the clear-sky atmosphere, coinciding with a symmetry of open ocean fraction, located at the 28° E meridian that hardly varies over the CERES observational record. This intriguing result is proposed to provide a benchmark with which climate models can be compared for realism and to gain understanding of processes driving energy balance and redistribution by ocean and atmospheric circulation. This result is novel; east-west hemispheric symmetries have not yet been investigated or demonstrated, and the newly found symmetries comprising more transient, dynamic climate system components (atmospheric circulation, clouds, and sea ice) and more fixed components (land/ocean fraction) is fundamentally interesting.

Overall, the manuscript is well-written, concise, and minimal, effectively delivering results from observational and climate simulation analyses. The abstract summarizes the finding effectively and concisely, and the introduction section nicely and efficiently introduces the work. The statistical and calculation methods used are sufficient to present the finding and compare it with climate models in a consistent framework. The data on which it is based are sufficiently robust and reliable, having provided the basis for most observational studies of Earth's radiation balance. Its graphics are high-quality and convey a good amount of interesting information in space-efficient but very readable ways.

My primary concern is about the justification of the finding's significance to climate studies, and the level of confidence in its significance. While I believe that the finding warrants publication and can provide insights into Earth's climate system, the manuscript could be improved by placing its findings in the context of existing work around spatial energetic contrasts like

the observed NH-SH albedo symmetry. This also concerns the effectiveness of the manuscript's conclusions section, which could benefit from this comparison so as to not overstate the significance of the findings, and to leave open the possibility that – just like the NH-SH albedo symmetry – the finding may be dependent on the time period considered, and may be affected by emerging changes in Earth's radiation balance beyond the period studied in the manuscript. Aside from this weakness, I believe the findings are significant, and the manuscript could be suitable for publication. Please see below for comments and suggestions that could help improve it.

Sincerely,

Aiden Jönsson

Major comment:

The authors introduce the topic by contrasting it to the NH-SH albedo symmetry (e.g. Stevens and Schwartz, 2012; Voigt et al., 2013; Stephens et al., 2015), referring to the recent publication suggesting that it may be breaking (Loeb et al., 2025), and demonstrating in the results how the E-W symmetry around 28° E persists strongly through the observational record. However, the period (through December 2022) on which this manuscript bases its finding coincides with the period in which NH-SH albedo symmetry is observed in the literature; Loeb et al. (2025) finds that it is primarily after this period that emerging asymmetry can be seen. This might beg the question as to whether or how soon we might expect a change in the reported E-W symmetry, which is not really explored in the manuscript. Extended Data Fig. 7 suggests an emerging trend in cloud asymmetries around 28° E. As the authors find about the E-W symmetry in different climate states in the model comparison section, the N-S albedo symmetry has been projected to change substantially as well (e.g. Rugenstein & Hakuba, 2023; Diamond et al, 2022).

Have the authors looked at the evolution of the E-W symmetry beyond 2022 to ensure that their results are not affected by the choice of study period? These data are available in CERES EBAF ed 4.2.1; is there a practical reason for not using data beyond 2022? The introduction frames an emerging NH-SH asymmetry by citing Loeb et al. (2025) and it is not certain that the E-W symmetry around 28° E is more robust or persistent; ensuring that the text reflects this caveat of the NH-SH albedo symmetry's evolution (perhaps best in the Discussion & conclusions section) could help the reader interpret both symmetries, and leave room to interpret the authors' findings in light of Loeb et al. (2025)'s findings.

Minor comments:

L44-48; Figure 1: I am not sure I understand how the variability in the longitude of E-W symmetry depicted in Fig. 1b is consistent with the claim of the 28° E symmetry meridian's robustness. As I interpret it, this figure shows that the average annual mean E-W difference around 28° E is little (and consequently the average of meridians landing at 28° E). However, the variability in symmetry meridians is therefore seemingly very large. Perhaps the manuscript could benefit from a supplementary figure with a PDF of symmetry meridians in the observational record, and the corresponding ΔR around 28° (as in Fig. 3b but replacing ΔO with ΔR), to judge the robustness of this claim.

L43: The interannual variability in ΔR around 28° E (the variation at 12 months averaging in Fig. 1c) compares reasonably well in magnitude to the interannual variability of the NH-SH symmetry (e.g. ±0.28 W/m2 reported in Datseris and Stevens, 2021; ±0.23 W/m2 reported in Jönsson and Bender, 2022), although it is slightly larger. This is what I assume manifests in the spread in meridians of symmetry in Fig. 1b. Stating this comparison in reported numbers here in the text could help the reader better judge the robustness of the newly found 28° E symmetry by placing it in the context of another symmetry that has been investigated in a number of studies.

L53-55, Figure 1a: Although the hemispheric differences in Rcld and Rclr at 28° E are small, there seem to be a few latitudes to the west where symmetry between these components are even stronger, only slightly impacting ΔR. It is hard to tell how much ΔR differs around these meridians, with the scales used in Fig. 1a and with the configuration of 1b; how much does it vary in this region? Since a point is made to call this a triple symmetry, it may be worth noting, while still emphasizing the point that the fact that it occurs near ΔO symmetry is striking.

Figure 3: Something seems off with the figure, or I am misunderstanding something; the caption refers to ΔR for (b), but the x axis is labeled ΔO. However, if it is ΔO, I do not understand how these values (spanning -0.2 to +0.2) are consistent with the values plotted in (a) (spanning -0.08 to +0.02). Are they both showing the E-W difference around 28° E along the x axis? If they are different, I suggest that the caption be adjusted for clarity.

Figure 3: The authors use single-realization model output to compare the observed E-W symmetry with, and do not characterize the variability in the E-W asymmetry around 28° E in these realizations (such as interannual, anomalies etc.). It could be helpful to include error bars (e.g. standard deviations in annual means) in the model mean asymmetries, because the ability or inability of models to capture the persistence that the authors found in the observed E-W symmetry would be an interesting finding, especially if it is suggested to benchmark models with. I am curious how it compares to the overestimated variability seen in modeled NH-SH albedo asymmetries across CMIP6 (Fig. 7 of Jönsson & Bender, 2023), which may tell us something about misrepresented processes in the models.

L162-165: The authors find a robust correlation between the ENSO phase and the E-W asymmetry around 28° E, which I find to be the strongest and most convincing argument that the E-W symmetry could be related to or even maintained by large-scale atmospheric circulation. The authors comment on the possibility that the E-W asymmetry in high vs. low clouds

could be related to this, which is a good lead for future studies and can point to a possible modulating mechanism. This is also reminiscent of the finding that the most extreme anomalies in the NH-SH albedo symmetry seem to reflect ENSO phase (Jönsson & Bender, 2023), which is an interesting facet of the results. The authors noted the positioning of the EH over the Western Pacific Warm Pool, which is certainly striking; I am wondering if the authors reflected on the relevance of the other rising branch of Walker circulation typically to the east of Central America, and how this affects? Is it that this is a primarily intrahemispheric variability in the WH where the associated high and low cloud variabilities compensate for each other, leaving little effect on the E-W symmetry? While not as prominent as the Pacific branch, it is curious how the EH dominance in high clouds/WH dominance in low clouds manifests considering they both have similar components in overturning (though with different spatial extents), and that ENSO/Walker circulation variability inevitably also alters cloud albedo outside of the Pacific.

Version 1:

Reviewer comments:

Referee #1

(Remarks to the Author)
The authors responded to my comments well and I do not have further comments on the new version.

(Remarks on code availability)

Referee #2

(Remarks to the Author)
I find the revised manuscript a vastly improved version of the original submission and recommend its publication. While I'm not sure this work will have enduring influence, it conveys an intriguing finding that will trigger debates within the ERB community immediately. Here are some additional comments on this version:

-- I'm very satisfied with the revised figure set. It's simpler, cleaner, and more intuitive.
-- I'm glad that Rcld was explicitly revealed to be CRE because that created great confusion to me (but why does Rcld still make a cameo since it is CRE that has now been adopted -- see caption of Fig. 1).
-- I agree with the thinking behind the statement that an interhemispheric link is likely more tractable for the E-W rather than N-S symmetry.
-- I see the title of the paper has changed now that the symmetry has shifted to 27°E from 28°E. This shows the perils of being so specific in the title. You add more years to the analysis, longitude may shift again. The analysis dataset is revised/processed you may shift slightly again. I suggest that the authors use a more general title to avoid this pitfall. Like "The Earth also has a longitudinal albedo symmetry"; "Earth's hitherto unknown East-West albedo symmetry". Something like that. Purely a suggestion.
-- So is this a "triple" symmetry or a "quadruple" symmetry? Because in Fig. 1 the triple symmetry is defined with the three black symbols (caption of Fig. 1a). But a fourth symmetry is also shown, namely the white color indicating equal area of ice-free ocean. The three black symbols only indicate a double symmetry because the quantities are not independent: DCRE=DR-DRclr. The nature of triple symmetry is correctly stated in the concluding section, lines 236-237.
-- Regarding the transient nature of the E-W symmetry and the parallels with the N-S symmetry: the equator is fixed, but not necessarily the longitude of the E-W symmetry. So when significant deviations from equal reflectance at 27°E start to appear does it simply mean that the longitude of equal reflectance has moved? Transient at a fixed longitude or transient in the sense of a moving longitude of equal reflectance?
-- OK, so the models don't have symmetry at 27°E. But do they have symmetry at another longitude? It'd have been interesting to say something specific about that other than their reflectance symmetry is not "necessarily at 27°E" (caption of Fig. 4).
-- Why are there no error bars on all values of Fig. 3?
-- I pointed this out previously, but the authors chose not to do anything about it: Why are hemispheric values added in extended Fig. 4? What is the physical interpretation of the sum? Please re-think this.
-- I like the waterfall plots a lot. Fig. 2b especially conveys a lot of information in a very immediate and concise way. But I wonder whether is problematic to include Extended Fig. 5a which is virtually the same as Fig. 1a of Diamond et al. (only difference is 95.8 vs 95.9 in SH reflectance -- all other numbers are the same). Why show a figure about N-S symmetry? I guess Ext. Fig. 5b is original...
-- Last line of Fig. 1 caption "are needed".

Lazaros Oreopoulos

(Remarks on code availability)

Referee #3

(Remarks to the Author)

The authors present a finding that the Earth can be divided into eastern and western hemispheres at the 27° E meridian with an intriguing degree of symmetry in clear-sky albedo, (shortwave) cloud radiative effect, and ice-free ocean fraction between them. This symmetry has a similar amount of persistence as the observed Northern-Southern Hemisphere albedo symmetry. The spread in simulated eastern-western hemisphere (a)symmetries around this meridian across GCMs is, like the N-S albedo symmetry, quite high. The E-W symmetry is, which may be expected given the zonal asymmetry of Walker circulation, highly correlated with the ENSO phase, and the authors also show that the E-W asymmetry in high/low cloud cover reflects this.

The revised manuscript feels more relaxed and realistic in its claims about the finding, which significantly improves it. There are a few cases where I reacted to strong language (see minor comments below), but I feel that the framing is honest and does not overclaim the significance of the finding. Moreover, the authors did significant work to situate the finding in the context of the N-S hemispheric albedo symmetry and compare between them, which I believe really helps the work.

Overall, the revised manuscript is very well written and thoroughly describes an interesting new finding certainly worth reporting! I am still somewhat uncertain about the true significance of the finding, but I believe the writing reflects that it is a surprising but open-ended discovery that may or may not lead to deep/useful constraints (just as the N-S albedo symmetry). While I leave it to the editor to decide if it is ultimately fitting for the journal, I am happy to say that the manuscript is essentially ready for publication, but offer a few minor comments below that may help improve it.
Minor comments:

L9: "... is a clarion call": I reacted to this quite strong wording, as I think there are other things more fitting as a "clarion call" (e.g. the total energy imbalance) when the authors also acknowledge the possibility that the reported discovery may be as coincidental as the N-S albedo symmetry.

L9, 11, 27, 59: The word "striking" appears many times and it feels a bit like it leads the reader rather than allows them to come to their own conclusions about the finding.

L103: "ocean&ice–land": This could do with some reformulation as it feels awkward, perhaps "ocean/ice-land" or "ocean-land" as the cryosphere is not commonly considered land cover.

L198: "... cooler sea surface temperature (SST) branch": I am a bit confused about which branch is being referred to? Of ocean circulation (and if so, where), or something else? If it is the subsiding branch of the Pacific Walker circulation, then "SST branch" is a bit confusing in atmospheric circulation contexts.

L228: "... low-degree-of-freedom": I do not really agree that this is a low-degree-of-freedom constraint. There are many ways to arrive at a given planetary albedo, e.g. through differing cloud fractions and optical depths, depending on cloud phase (e.g. Crueger, Schmidt & Stevens, 2023). This "compensating error" feature has been described as a reason for the limited ability of the N-S albedo symmetry to constrain cloud feedbacks (Jönsson et al., 2025) and climate sensitivity (Jönsson & Bender, 2023). While it does remove a few variables compared to the N-S albedo symmetry, the large spread in modeled asymmetries around 27° E hints at a wide range of albedo distributions resulting from differing modeled processes. Would the authors be able to substantiate/elaborate on the claim that it could be a low-degree-of-freedom constraint? If not, I would suggest that this claim also be relaxed.

References:

Crueger, T., Schmidt, H., & Stevens, B. (2023). Hemispheric albedo asymmetries across three phases of CMIP. Journal of Climate, 36(15), 5267-5280. DOI: 10.1175/JCLI-D-22-0923.1

Jönsson, A. R., & Bender, F. A. M. (2023). The implications of maintaining Earth's hemispheric albedo symmetry for shortwave radiative feedbacks. Earth System Dynamics, 14(2), 345-365. DOI: 10.5194/esd-14-345-2023

Jönsson, A., Rugenstein, M., Bender, F. A. M., McCoy, D., & Eidhammer, T. (2025). Parametric sensitivity of hemispheric albedo symmetry weakly constrains shortwave cloud radiative feedbacks in the Community Atmosphere Model version 6. Geophysical Research Letters, 52(14), e2025GL115948. DOI: 10.1029/2025GL115948

(Remarks on code availability)

Open Access This Peer Review File is licensed under a Creative Commons Attribution 4.0 International License, which permits use, sharing, adaptation, distribution and reproduction in any medium or format, as long as you give appropriate credit to the original author(s) and the source, provide a link to the Creative Commons license, and indicate if changes were made.
In cases where reviewers are anonymous, credit should be given to 'Anonymous Referee' and the source.
The images or other third party material in this Peer Review File are included in the article's Creative Commons license, unless indicated otherwise in a credit line to the material. If material is not included in the article's Creative Commons license and your intended use is not permitted by statutory regulation or exceeds the permitted use, you will need to obtain permission directly from the copyright holder.
To view a copy of this license, visit https://creativecommons.org/licenses/by/4.0/

Response to reviews of "Earth's albedo symmetry at 28°E" by J. Zhang et al.

We would like to thank the editor and the three reviewers for their insightful feedback and constructive comments and suggestions on our manuscript, which have helped us to improve the original manuscript. First, we want to highlight key updates to the revised manuscript, as suggested by the reviewers' and editor's comments:

1. **We switched to EBAF for the majority of the analysis, to avail ourselves of its longer record and more complete diurnal filling.** In a nutshell, the majority of the analysis uses EBAF 2001-2024; for cloud type decomposition, FBCT 2003-2022 is used; and for recent trends in ΔR, EBAF 2001-2025 is used. We note that both EBAF and FBCT yield a robust identification of the E-W symmetry, but the dividing meridian is **27 E** in EBAF and **28 E** in FBCT, reflecting different stream processing associated with these two CERES products (see **Fig. R2** for a proof of robustness and consistency between the two records and **L272-276** in the revised manuscript for a mention of this difference). All results and discussions are updated, reflecting this change.
2. **We streamlined the discussion** around how the E-W symmetry is defined and referred to throughout the revised manuscript, resulting in changes to the old Fig. 1 and Fig. 3.
3. **We have clarified that 'Rcld' represents 'cloud radiative effect (CRE)'.** Neglect of this explicit association in the original submission caused a key confusion to reviewer#2. We now use 'CRE', instead of 'Rcld', throughout the revised manuscript.
4. **We now include a total of 8 ESMs from CMIP6 (increased from 5)**, representing one from each model family, preferably with participation in the GeoMIP experiment. The rationale for model selection is added.
5. **We updated the surface classification to a time-varying classification**, instead of the climatology-based classification, to better resolve the emerging changes in Earth system components.
6. **We now, throughout the revised manuscript, discuss the discovery of E-W symmetry in the context of the N-S symmetry**, including a potential connection between the two albedo symmetries and their persistence and trends.
7. **Figs. 1-4 have been reconstructed** to reflect these above-mentioned major changes.

We have also made unsolicited updates and changes to the manuscript for clarification and readability, as captured in a tracked changed document.

Specific point-by-point responses to each comment are contained below, with the reviewers' comments provided in **blue** and our responses in **black**. Changes to the manuscript made in response to the reviewer are provided in ***red italics***.

Referee #1:

Review of a paper by Zhang et al. entitled "Earth's albedo symmetry at 28 degree E" submitted to Nature.

The authors found that the 28-degree east meridian divides eastern and western hemisphere with an equal annual mean open water fraction. In addition, the eastern and western hemisphere have the same clear-sky and cloudy sky albedos. This implies that cloud contributions to the albedo for the eastern and western hemispheres are the same. Because the western hemisphere contains marine stratocumulus regions, this also implies that high clouds in the eastern hemisphere compensate a smaller contribution from low level clouds in the eastern hemisphere. Furthermore, in the last 20

years, the western hemisphere is darkened due to decreased cloud amount while the eastern hemisphere is darkened due to decreased land albedo. The authors also found a correlation between albedos of cryosphere and lower-latitude land regions. The authors investigated whether climate models can produce the eastern-western hemispherical albedo symmetry. While most of the tested models fail to produce the symmetry, the bias of the symmetry seems to be caused by the bias of maintaining the symmetry of open water area. For the cloud contribution to the symmetry, the authors found that only the GISS model maintains the cloud contribution symmetry.

The eastern-western albedo symmetry that the authors discovered is interesting. Also, the manuscript is well written and discusses authors' thoughts on the symmetry. However, the manuscript does not explain beyond this curious nature of the symmetry. The community accepted that the northern hemisphere and southern hemisphere albedo symmetry is a property of the current climate system. Whether this property is the intrinsic nature of the climate system and holds for the future climate are debatable. Given that, the manuscript provides an open-ended question of whether the albedo symmetry is one of intrinsic properties of the climate system. What is missing in this manuscript is, however, to link the eastern-western hemispheric albedo symmetry to the northern-southern hemispheric albedo symmetry. It is possible that two albedo symmetries are caused by completely different reasons, but the authors can apply similar analyses to both symmetries. For example, performing a similar analysis shown in Figure 2 to northern-southern hemispherical hemispheres might give us a clue of fundamental requirements for the symmetry.

We thank the reviewer for the kind words and insightful comments. The reviewer raised a really important point, which motivated us to dig deeper into the potential connection between N-S and E-W symmetries. In the revised manuscript, we now discuss the connection between the two hemispheres throughout the main text within each section. For the revised Figs. 2 and 3, the same analyses have been done for the N-S symmetry and added as Extended Data Fig. 5 and 8 to facilitate the discussion. Moreover, we have also included a new Extended Data Fig. 9, where we show how models capture N-S and E-W symmetries as a scatter plot, and in the revised Fig. 1c, we show a direct comparison of the interannual variability between the two symmetries. The revised manuscript now offers deeper thoughts on the observed Earth albedo symmetries (N-S and E-W) and conveys a high-level message on the value of this E-W symmetry discovery: *The importance of the E-W symmetry discovery, however, is beyond the identification of another "sweet spot" of the Earth system: it offers a powerful, low-degree-of-freedom constraint on state-of-the-art ESMs, and, more broadly, on our fundamental understanding of the Earth climate system. This is because its distinctive "triple symmetry" feature and connection to ENSO extend the observational constraint beyond TOA all-sky reflection to the coupled behavior of clouds, the ocean, and sea ice in models.*

Changes are made throughout the revised manuscript to convey this point as follows.

"L41-43: Although there is little evidence for a direct connection between the N-S and E-W symmetries, they are both on trajectories toward a departure from symmetry in response to emerging changes in Earth's radiation balance over recent years \cite{Loeb25,Oreopoulos25,Singer25}"

In the section 'Observed "triple symmetry" at 27° E':

"L71-73: We observe similar magnitudes of interannual variability in ΔR between the E-W symmetry (at 27o E; ±0.24 W m-2) and the N-S symmetry (at the equator; ±0.20 W m-2)[2,7] (Fig. 1c)."

In the section 'Compensation between low and high clouds maintains the E-W symmetry':

"L104-113: A similar compensation among different surface types is found for the N-S symmetry (Extended Data Fig. 5b), but it is much larger in magnitude and arises mainly from the strong land-ocean contrast between the NH and SH (Extended Data Fig. 2b). Consequently, the N-S symmetry is maintained largely by stronger CRE in the SH (mostly from low-level clouds) compensating for stronger clear-sky reflections in the NH (Extended Data Fig. 5a and[10]). Thus, although TOA all-sky SW reflection is climatologically split equally between the two hemispheres, decomposition into reflection components reveals that the N-S and E-W symmetries are maintained through very different hemispheric compensations. Moreover, the corresponding hemispheric contrasts in clear-sky reflection, CRE, and land-ocean fractions at these all-sky reflection symmetries also differ substantially (Fig. 1a and Extended Data Fig. 5), suggesting that the two albedo symmetries are likely not directly connected. Given the record global mean temperatures of the past few years, the question arises: are the distinct compensations that maintain the N-S and E-W symmetries being sustained amid emerging changes in the Earth system?"

In the section 'Recent trends in hemispheric albedo symmetries':

"L141-151: The N-S contrast in these emerging Earth system changes over the past two decades (Extended Data Fig. 8) is expressed primarily through clear-sky atmospheric reflection: darkening in the NH from anthropogenic aerosol clean-up and brightening in the SH from enhanced natural aerosol production over the Southern Ocean[13]. Consequently, the N-S asymmetry trend derived from the 2001-2024 EBAF record is statistically significantly different from zero (gray filled marker and error bar and in[11]). Despite the apparent lack of a direct connection between the two hemispheric symmetries, both appear to be departing from symmetry as Earth undergoes substantial environmental change. This suggests that both symmetries may be transient features of the present climate system, reflecting the same non-stationary Earth system through different lenses. Intriguingly, despite the continuation of record-breaking global mean temperatures in 2025, both E-W and N-S ΔR shift back toward symmetry when 2025 is included in the trend calculations (gray filled squares with dashed outlines in Fig. 3 and Extended Data Fig. 8, and the time series in Fig. 1c). The cause of this reversal warrants further investigation, potentially involving outgoing longwave radiation[11, 12]."

In the section 'Earth system models diverge and suggest a non-stationary E-W symmetry':

"L186-188: Overall, these simulations lend further support that the E-W and N-S asymmetries do not appear to be directly connected (Extended Data Fig. 9). In most models, the N-S asymmetry shows larger shifts across simulated periods and scenarios than the E-W asymmetry."

In the section 'The E-W albedo symmetry is connected to ENSO variability':

"L208-215: This shift in the Walker circulation could trigger cascading, potentially cross-equatorial changes in cloud distribution through coupling with meridional overturning circulations. Indeed, the

most extreme interannual anomalies in the N-S symmetry have been linked to ENSO phases[7]. Our analysis of the N-S symmetry suggests a weaker and insignificant correlation between ENSO variability and the interannual variability of the N-S symmetry (r=-0.33, p=0.11). This is consistent with the fact that ENSO is largely a mode of variability in the zonal direction. That the observed hemispheric albedo symmetries exhibit different degrees of connection to ENSO suggests they essentially capture the "pulse" of the same climate system in distinct ways. Therefore, potential alteration to atmospheric overturning circulations such as the Walker circulation under climate change[38, 39] could lead to a breakdown of Earth's albedo symmetries."

In the section 'Discussion and Implications':

"L225-227: Similar to the N-S albedo symmetry, one cannot yet rule out the possibility that these hemispheric symmetries are simply coincidental features of the current climate state. In fact, accumulating evidence suggests that this may be the case[11–13], including the findings of this work."

I understand that the eastern-western hemispheric albedo symmetry is different from northern-southern hemispheric albedo symmetry because the former has the same clear-sky albedo while the northern hemisphere has a larger clear-sky albedo. However, clouds compensate darkening land in the former symmetry might provide a clue to the latter. I believe that the linking these two albedo symmetries is important for this manuscript so that it makes this finding transferring from a merely curious finding to investigating intrinsic properties of the climate system. In doing so, I suggest the authors to come up with a working hypothesis for requirements of the albedo symmetry, justifying the motivation of finding eastern-western hemispherical albedo symmetry (probably right before line 22), and testing the hypothesis. Although some of authors' thoughts are given in the current version, making a hypothesis and justifying finding of the eastern-western hemispheric albedo symmetry makes the manuscript flow better. If, however, the authors argue that the eastern-western hemispheric albedo symmetry is not related to northern-southern hemispheric albedo symmetry, please justify the scientific motivation of finding the symmetry and describe conclusions accordingly.

We appreciate this comment; it has motivated us to improve the overall storyline of this work. In light of the reviewer's suggest, we formulate a working hypothesis, illustrate what our findings on the E-W symmetry imply about the hypothesis, and discuss interpretations of the findings. This change has been incorporated throughout the revised manuscript and summarize below. In the revised storyline, we address this working hypothesis at each section, with the goal of maintaining a wide scope of the study while following a hypothesis-based storytelling.

1. **Working hypothesis:** Similarities or differences across symmetry pairs could shed light on the underlying couplings among Earth system components.
2. We discovered an E-W symmetry at 27 E [Fig. 1] and compared it with the N-S symmetry:
   a. The two symmetries are maintained by fundamentally different hemispheric compensations, suggesting <u>no direct connection</u> between the two. [Fig. 2]
   b. Observed trends in both symmetries indicate a tendency toward departure from symmetry, suggesting albedo symmetry may be a <u>transient</u> feature of the current climate, <u>each as an independent manifestation of the same system</u>. [Fig. 3]

c. CMIP6 simulations indicate little connection between the two but suggest they are both non-stationary and susceptible to shift under different climates. [Fig. 4]
d. Interannual variability of the E-W symmetry is found to be strongly connected to ENSO phases, suggesting an important role of the Walker circulation in helping maintain the E-W symmetry. Similar, albeit weaker, connection between N-S symmetry and ENSO has been noted in earlier work (Jönsson & Bender, 2022), reinforcing the interpretation that the seemingly independent symmetries could be connected indirectly [Fig. 5]

3. These findings suggest that the two symmetries are likely not connected. They lend support to the interpretation that the two albedo symmetries are nonstationary, transient features of the current climate system that may break down in future climates. Nevertheless, the connection between E-W symmetry, as manifested in the triple symmetry, and atmospheric circulation represents a great opportunity to learn more about the compensating mechanisms within the Earth system.
4. We end with an emphasis on the critical role of the continuation of Earth Radiation Budget measurements to validate this interpretation of the two symmetries.

This working hypothesis is now posed at L21: *"Similarities or differences across symmetry pairs could shed light on the underlying couplings among Earth system components."*

Minor comments

Figure 1: the waterfall chart is confusing. Could you explain what the gray bars represent? Also, it is not clear how values on the y-axis are related to other color bars. If the y values are not related with colored bars, I suggest using a simple bar chart.

We assume the reviewer is referring to the waterfall chart in Fig. 2. The idea of using a waterfall chart to illustrate compensations across Earth system components between the two hemispheres was first developed in **MD24**. This has been noted within the community as an effective visualization to illustrate the cumulative rise and fall of component contributions to hemispheric differences, showing the transition back to the same reflection magnitude in the opposite hemisphere. We nonetheless agree with the reviewer that some initial effort is required to interpret the chart before its effectiveness is fully realized. To help the reader better understand this chart, we have added details to the figure caption in the revised manuscript. Essentially, the gray bars simply represent the climatology (24-year mean) of hemispheric SW reflections, which are nearly identical between the two hemispheres, indicating the symmetry. The colored bars show the differences (EH minus WH) in each component (denoted by the color-hatching combination as illustrated by the insert) between the two hemispheres.

The Fig. 2 caption has been updated as follows: *"**(a)** Waterfall chart showing hemispheric compensations across components of R (colored bars), with positive (negative) values of the colored bars denoting greater EH (WH) reflection. EH and WH are demarcated at 27o E (see main texts). R is decomposed into contributions from surface (sfc) under clear-sky, atmosphere (atm) under clear-sky, and the cloud radiative effect (CRE) for different cloud types (illustrated by the insert), based on the CERES FluxByCldTyp (FBCT) product for 2003-2022 (inclusive). Gray bars indicate the hemispheric-*

mean R (R), which is nearly identical between EH and WH. A clear compensation between the low-level clouds (more reflective in the WH) and high-level clouds (more reflective in the EH) is evident."

Line-138-140: Because the fitted magenta line goes through the origin in Figure 3a, does this imply that if a climate model were to have the correct open ocean area symmetry, it would have modeled the albedo symmetry correctly? In other words, does the failure of modeling albedo symmetry come from the failure of modeling sea ice symmetry?

We realize this regression might actually mislead the interpretation of this figure, and thus it was removed in the new Fig. 4. The new Fig. 4 now present the model results in a more straightforward way. Both in the original Fig. 3a and new Fig. 4a, models actually capture the observed ΔO at 27 E fairly well. Therefore, we believe the failure of the modeled albedo symmetry is due to the deficiency in representing the coupling across Earth system components, e.g., sea ice and clouds over lower latitudes. We have added a discussion to clarify this:

"L160-163: Overall, all models reproduce the observed ΔO (color of the bars and markers) at 27o E reasonably well, indicating a good representation of sea-ice dynamics, with the exception of MIROC6. This suggests that although variability in sea-ice is relatively well captured, its coupling to the other Earth system components is not well represented in these models."

Figure 3: I am puzzled by the differences of the x-axis values of data points between figure 2a and 2b. If Figure 2b replaces the y-axis of Figure 2a by longitude, I expect that all points in Figure 2b have the same x values with Figure 2a.

Thanks for raising this point. We realized the presentation of this figure could be confusing and overwhelming. In the revised Fig. 4, we adopted a much more straightforward way to show the model results, with everything related to the E-W symmetry shown at 27 E and departures from symmetry shown as W m^{-2} differences, instead of longitude differences.

Methods: When computing hemispherical means, are 1 degree by 1 degree areas weighted by the area?

Yes. This was stated in the 'Spatiotemporally weighted averaging, statistical significance, and trend analysis' subsection of *Methods*. We now emphasize it with a slight rewording:

"L335-336: In calculations of hemispheric, regional, zonal, and global climatologies, spatially and temporally weighted averaging is performed, taking into account the difference in grid box size, month length and the geometry of the Earth as an oblate spheroid."

Referee #2:

The fact that the Northern and Southern Hemispheres reflect approximately equal amounts of solar radiation has been a widely documented and studied topic, although no definitive explanation has

so far been given as to why this state of affairs should exist. Actually, two of the three authors of this study have co-authored a paper that examined aspects of this NH-SH symmetry, namely in what this manuscript labels as citation [9], wherein some figures, like the waterfall plots, look exactly like the ones here. In this study, the authors present on the discovery of a previously unknown symmetry in the way the planet reflects sunlight. Two hemispheres are again involved, but this time the demarcation line is not the equator, but the 28°E meridian, in a climatological sense: in short, instead of northern and southern hemispheres, now we are dealing with eastern and western hemispheres (EH and WH) reflecting approximately equal amounts of sunlight. Contrary to the N-S split for which the symmetry makes sense at temporal scales of a year or multiples thereof (with some adjustments to account for subtle differences between leap and non-leap years, see Material and Methods in citation [10]), because of the seasonal dependence the insolation that the N-S hemispheres receive, the E-W split can in principle be examined at scales as short as a day and multiples thereof from an insolation symmetry perspective. Curiously, this important distinction is not mentioned in the manuscript. While Fig. 1c in [9] didn't make sense to me, the same figure (reoriented) in this manuscript, also Fig. 1c, now makes total sense. Along the same lines, extended Fig. 5 does not make sense for the N-S symmetry, but is entirely legitimate for the E-W symmetry. It is important to emphasize a point that may be missed by a superficial examination of the paper: the 28°E meridian is the median of longitudes where symmetry of reflected fluxes between EH and WH occurs as monthly values of reflectance are being accumulated; for individual years (and presumably shorter time scales) a different meridian defines the equally reflecting hemispheres (Fig. 1b).

So is this (well-written and organized) paper worthy of publication in Nature, provided some issues are addressed? It is a tough call. I am leaning no for Nature proper specifically, but open to change my mind, and am certainly positive to publications of lesser stature. A compelling new (as far as I know) finding is documented here about which the authors appear to be genuinely excited; but like the NH-SH symmetry, it remains more of a curiosity without a plausible physical link to the workings of the Earth System. It should be noted that while there may be no explanation for why the mean state of the Earth has symmetric reflectance around 28° (so it could be fortuitous), it seems that deviations from symmetry (more on that later) are linked to ENSO which affects the Walker circulation which in turn affects the meridional distribution of cloudiness. This is one of the important findings of the paper (Fig. 4) that could serve as springboard for further studies.

I have both major and minor points on certain aspects of the analysis and the presentation of the results. Not sure whether the major ones will trigger revisions in the calculations, but I want to help ensure that something didn't go amiss in the calculations.

We thank the reviewer for the kind words and insightful comments!

Regarding the comment "the E-W split can in principle be examined at scales as short as a day and multiples thereof from an insolation symmetry perspective. Curiously, this important distinction is not mentioned in the manuscript" that wasn't reiterated below, we have added a paragraph in the revised manuscript to address this.

"L98-103: Another distinct feature of the E-W symmetry is that, theoretically, unlike the N-S symmetry, it does not require a full annual cycle to achieve an equal amount of SW reflections from the two hemispheres, given the revolving nature of the Earth in the E-W direction. We found that at seasonal timescales the E-W symmetry and the compensation across its components generally break down, mainly due to the strong seasonal variability in cloud radiative effect (Extended Data Fig. 4). The hemispheric differences in CRE at seasonal timescales range from +3.5 W m^{-2} (June-August) to -2.9 W m^{-2} (September-November). We note, however, that the ocean&ice—land compensation pattern is largely maintained during boreal winter and spring."

Major points

1. I think it is critical that the interannual variability and trends are discussed with maximum possible clarity. The 28°E meridian is a median longitude (L47) of different longitudes defining EH and WH with equal solar reflected fluxes, right? First of all why are you defining this "golden" meridian this way? Why not find the meridian that makes the 20-year climatological reflected fluxes of two hemispheres equal? In any case, the bottom line is that for individual years equality is achieved at different longitudes (Fig. 1b). In other words, for individual years there is in general no symmetry at 28°E. So what is then the meaning of the trend in the (a)symmetry in figures such as Fig. 1c? Is 28°E fixed and a linear fit is applied to those deviations from symmetry (L95-96)? You can always find symmetry for individual years as long as you move the meridian, so the trend is then zero for a moving meridian. But I guess then that the trend shown in the thick black line of Fig 2c comes from a linear fit to the blue line of Fig. 4?

We appreciate these comments and questions as they reflect a confusion that arose from the way we discussed the E-W symmetry, which is the same reason why reviewer#1 was puzzled by our original Fig. 3.

First of all, the way we find and define the 27 E meridian as the E-W symmetry was exactly as the reviewer suggested, that is, the meridian that "makes the 20-year climatological reflected fluxes of two hemispheres equal". Although the median of different longitudes defining EH and WH with equal solar reflected fluxes also converges to 27 E, we did not define the E-W symmetry this way. We have revised the text to make this point clear. Because one can always find a symmetry at different longitudes for individual years, a key difference from the N-S symmetry, the interannual variability of the E-W symmetry can be expressed either as a longitude departure from the climatology-27 E or as the departure of the hemispheric flux difference from zero at 27 E. We realize that this was a key source of confusion. Therefore, we choose to use only one expression (the latter) throughout the revised manuscript: we always assess the E-W symmetry at 27 E (the climatologically defined meridian) and express departure from symmetry as the departure of hemispheric flux difference from zero. Regarding the trend calculations, as mentioned above, we calculate the hemispheric flux difference at the 27 E meridian. So, the reviewer's understanding is correct.

We have streamlined the discussion around these points in the revised manuscript and have re-constructed Figs. 1-4. As part of the figure reconstruction, we added an assessment on the robustness of the 27 E meridian as the climatologically defined E-W symmetry (Fig. 1b).

“L51-54: A hemispheric symmetry in R is identified at 27o E (ΔR of -0.04 ± 0.24 W m-2) based on the 25-year (2001-2025, inclusive) EBAF record (see Methods). Therefore, we henceforth use 27o E as the reference meridian for EH and WH and for assessing the degree of asymmetry in interannual variabilities and in climate models.”

2. I don’t understand R=Rclr+Rcld at any spatial scale/domain and either of the two CERES products, FBCT or EBAF. If R is the all-sky TOA flux, shouldn’t it be R=(1-CF)Rclr+CF*Rcld which is consistent with the definition of CRE=R-Rclr=CF(Rcld-Rclr)? There has to be weighting in the summation, unless the clr and cld fluxes have already been pre-weighted which is not stated anywhere as far as I can tell. See eq. (6)-(8) in citation [6]. While FBCT gives you Rcld, and also its breakdown by cloud type, EBAF gives only all-sky R and clear-sky Rclr. But you can certainly not get the gridbox flux in FBCT by just summing Rclr and Rcld; you have to weight by the area of the clear and cloudy portions. By the same token you can’t get Rcld as simply the sum of cloud fluxes of cloud types (L224), you have to take weighted average by cloud type population. I’m not sure whether this simply a matter of these relationships being shown symbolically rather than explicitly and rigorously or the calculations being done incorrectly. This is certainly merits clarification, especially when other types of weighted averaging (like accounting for different number of days in a month) are documented.

We thank the reviewer for pointing out this important detail that we neglected to mention when describing the decomposition. Indeed, the cloud fraction weighting was already applied when calculating the decomposed R values. We now explicitly state that the R values are pre-weighted by cloud fraction. Moreover, we think the confusion is rooted in our neglect to mention that ‘Rcld’ refers to CRE in this study. We therefore use CRE, instead of ‘Rcld’, throughout the revised manuscript.

As mentioned, the notation ‘Rcld’ in our original manuscript is succinctly referring to CRE, which is defined as ‘R - Rclr’. For EBAF, the CRE for all clouds is straightforwardly calculated as the difference between R and Rclr; for the CRE of different cloud types in FBCT (i.e., CRE_{type}), we calculate each CRE_{type} weighting by the cloud fraction of each cloud type as ‘CF_{type}*($Fcld_{type}$ - Fclr)’ where CF_{type} is the cloud fraction, $Fcld_{type}$ is the upwelling overcast TOA flux for that cloud type, and Fclr is the upwelling clear-sky TOA flux from FBCT.

These details are now added to the Methods, and discussion around “cloud reflection” has been streamlined and clarified throughout the revised manuscript.

“L282-285: The cloud contribution to the total all-sky TOA SW reflection is defined as the cloud radiative effect, calculated as the difference between all-sky reflection (R) and clear-sky reflection (for the total region; Rclr). The CRE is then further decomposed into contributions from each cloud type, calculated as the cloud fraction (fcldtyp) weighted flux difference between upwelling overcast TOA flux for that cloud type (F ↑cldtyp) and the upwelling clear-sky flux (F ↑clr): Rcldtyp= fcldtyp(F ↑ cldtyp−F ↑clr)”

3. How do you get a gridcell's Rcld, which I interpret as overcast cloud flux, from a climate model? Again, to my knowledge, a model gives you only R and Rclr. Unless you obtain it through the model’s

vertically projected planar cloud fraction, CF. But CF is not a variable that appears anywhere in the manuscript, in contrast to [6].

As mentioned above, we realized that this question may arise from our neglect of explicit association between 'Rcld' and CRE. Similarly to EBAF, the 'Rcld' (or CRE) in CMIP6 ESMs is calculated straightforwardly as the difference between 'rsut' and 'rsutcs', that is 'TOA Outgoing Shortwave Radiation' minus 'TOA Outgoing Shortwave Radiation assuming Clear-Sky'. We have clarified this in the revised manuscript as well. As mentioned in the above response, we now use CRE, instead of Rcld, throughout the revised manuscript.

"L325-327: We obtain 'rsut' (TOA all-sky SW reflection), 'rsutcs' (TOA all-sky SW reflection assuming clear-sky), and 'siconc' (sea ice area fraction) variables from these model simulations. TOA flux decomposition is calculated in the same way as in the CERES products (CRE = rsut - rsutcs)."

4. Why is only Rclr decomposed to surface and atmosphere contributions? In cloudy columns, Rcld does not come only from the cloud alone (the terminology is not exact throughout the paper, it's the reflectance of the cloudy column rather than the reflectance of the cloud), but from the combined contributions of cloud, surface, and (cloudless) atmosphere. When the cloud is of small or moderate opacity over a land or ice surface (two situations explicitly resolved), the surface contribution can be substantial. The decomposition of reflected flux as performed in the paper is therefore incomplete in my opinion. If nothing is done about it, this should at minimum be identified and discussed.

Again, we believe this concern is also related to our neglect of explicit association between 'Rcld' and CRE. CRE, as discussed above, is calculated by removing the clear-sky reflection from the all-sky reflection with consistent surface properties, leaving only cloud contributions to the reflected fluxes. As mentioned in the above responses, we now use CRE, instead of Rcld, throughout the revised manuscript.

5. Isn't the enthusiasm about the skill of the GISS model in reproducing the E-W symmetry tempered by the fact that the absolute values of reflected flux are so biased? PD values of around 103 Wm-2 (extended Fig. 8c), corresponding to a planetary albedo of 0.30, compared to ~96 Wm-2 in observations (Fig. 1a,b, presumably FBCT), corresponding to a planetary albedo of 0.28, and ~99 Wm-2 from EBAF (e.g., Oreopoulos et al. 2025), corresponding to a planetary albedo of 0.29 (the value quoted in the paper)? 4 and 7 Wm-2 are huge biases! I would probably trust more a climate model that gives me correct current state planetary albedo than one that gives me the most symmetry around 28°E.

The reviewer has raised an important point and we fully agree the mean state bias in hemispheric fluxes warrants caution about the fidelity of the simulation. In fact, in the revised manuscript, we have switched to GISS-E2-1-H instead of the GISS-E2-1-G, because the ocean model in GISS-E2-1-H has finer spatial resolution. As a result, the hemispheric difference in open ocean fraction (delta_O) better matches the observation in GISS-E2-1-H, but it no longer captures the observed E-W symmetry. In addition, we have included 3 additional models from CMIP6, CanESM5, CESM2-WACCM, and MPI-E2-1, a total of 8 ESMs, one from each distinct model family (Kuma et al. 2023 JAMES). They are

selected based on their participation in both historical and SSP3-7.0 experiments with a preference given to the model that also participated in the GeoMIP (specifically G6sulfur) experiments. In the revised manuscript, we have refrained from interpreting the fidelity of a particular model, but rather discuss them as a group, such as:

"L159-163: ESMs' present-day (PD, 1995-2015) simulations, as part of CMIP6's historical experiment, diverge at the observed E-W symmetry of 27o E (bars in Fig. 4a), ranging from -2 W m^{-2} (MPI-ESM1-2) to 5.2 W m^{-2} (MIROC6). Overall, all models reproduce the observed ΔO (color of the bars and markers) at 27o E reasonably well, indicating a good representation of sea-ice dynamics, with the exception of MIROC6. This suggests that although variability in sea-ice is relatively well captured, its coupling to the other Earth system components is not well represented in these models."

"L163-166: Another important result from these simulations is that, although they fail to capture the observed E-W albedo symmetry, nearly all models show a similar shift in ΔR from pre-industrial (PI) to the "regional rivalry" socioeconomic Pathway (SSP3-7.0) at the end-of-century (EOC), with GISS-E2-1-H as the exception (markers in Fig. 4)."

"L169-171: The four G6sulfur simulations (magenta markers), representing a climate intervention scenario in which sulfate aerosols are injected into the stratosphere to scatter sunlight and cool the planet—a proposal known as Stratospheric Aerosol Injection (SAI), further show that large perturbations to the Earth system can alter the E-W ΔR."

6. Have the authors considered examining (a)symmetry around 28E of other energy components besides reflected flux? OLR, the atmosphere's radiative cooling? How close or far are the two EH or WH in radiative convective equilibrium (if this something that matters in explaining the symmetry phenomenon)? Other energy components are examined in N-S symmetry papers, for example in Oreopoulos et al. (2025) and citation [10].

Yes, we have. OLR and EEI have very different symmetry longitude that are both very persistent with weak interannual variably (**Fig. R1**). We agree this analysis can definitely help us better understand the E-W symmetry, but given the limited space in the journal format, we choose to stimulate follow-on studies that address this front.

"L147-151: Intriguingly, despite the continuation of record-breaking global mean temperatures in 2025, both E-W and N-S ΔR shift back toward symmetry when 2025 is included in the trend calculations (gray filled squares with dashed outlines in Fig. 3 and Extended Data Fig. 8, and the time series in Fig. 1c). The cause of this reversal warrants further investigation, potentially involving outgoing longwave radiation \cite{Loeb25,Oreopoulos25}."

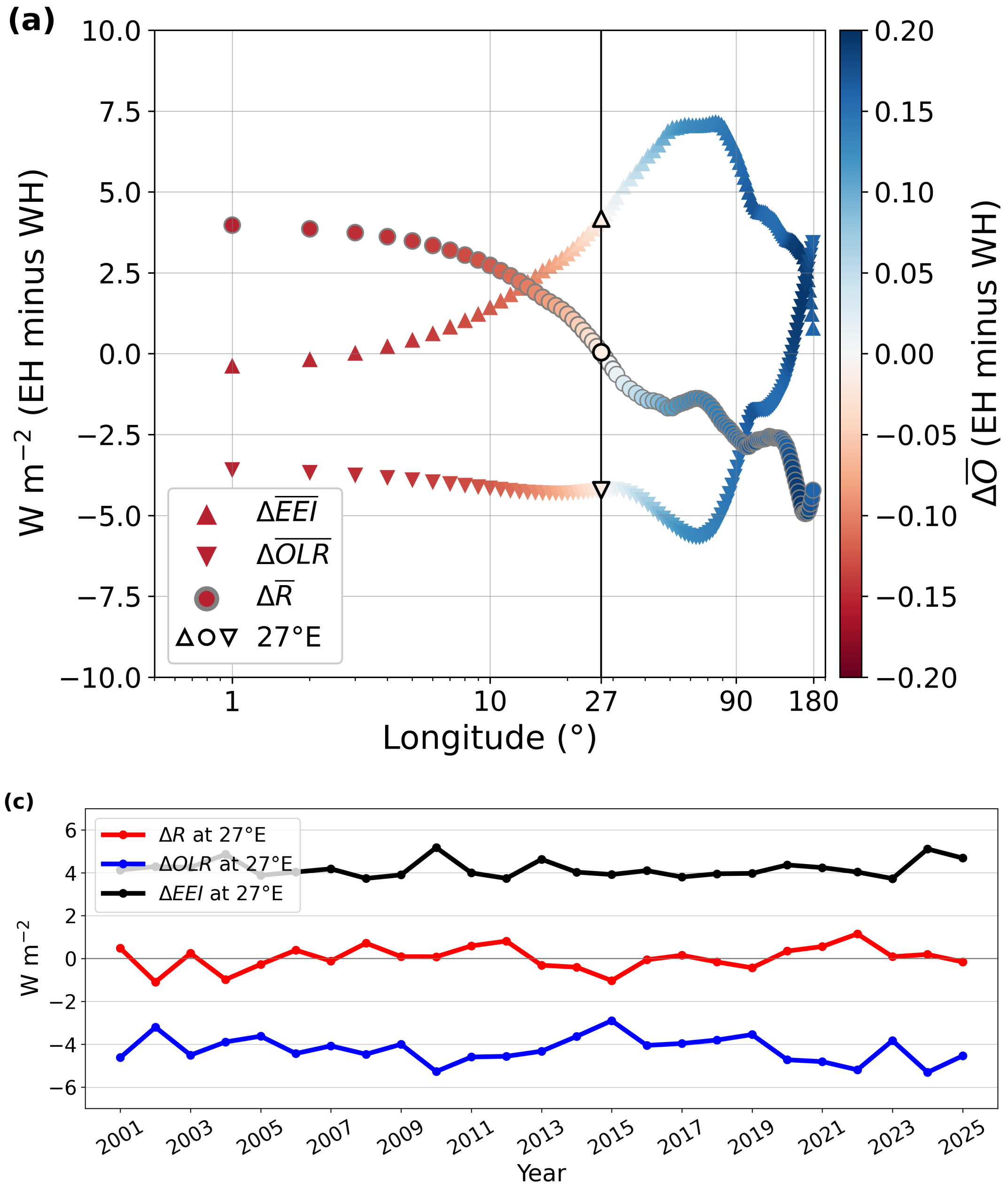

Figure R1. As in Fig. 1a and c, but for OLR and Earth Energy Imbalance (EEI).

7. In the lag analysis, are the authors basically implying that there are processes involving clouds that operate on the scale of a full year?

We do not go as far as stating that cloud processes are operating on the scale of a full year. The purpose of this lagged correlation analysis is to identify a potential causal connection among components of the Earth system. That being said, we realize that only speculative information can be gained from this analysis, we have removed it from the main text and only show two statistically significant ones in Extended Data Fig. 7.

"L139-140: A lagged correlation analysis based on interannual variabilities in ΔR and its components, provided in Extended Data Fig. 7 and its caption, has identified several potential couplings for future exploration."

Miscellaneous points of wide-ranging importance

1. Abstract: Why is the albedo symmetry "delicate"? In what sense? "Each of which ALSO persistently". Why "also"? What else do these hemispheres do? You haven't said yet that they have equal land-ocean ratios. "reflectS" instead of "reflect". "at this meridian" or "around this meridian"?

We thank the reviewer for these comments. The abstract has been rewritten incorporating these points.

2. Lines 17-21: 3% is still more than what you find here (n=1), no? My understanding is that the areas making the equal hemispheres in the cited study were allowed to be non-contiguous. So your finding is much more special!

Thank you! This is exactly why we reference this work to set the stage for our finding that there is a single solution in longitude that divide the Earth into non-overlapping pairs is striking. We have specifically emphasized this point in the revised manuscript.

"L26-28: The finding of the E-W symmetry at 27o E is striking for its persistence and the fact that it represents the only longitudinal divide yielding such balance."

3. L35-36: "symmetry in cloud reflection". See my major point (4). Cloud reflectance may not be symmetric, but the contributions from everything else in the cloudy column make it symmetric. See also L85-86.

As mentioned in the responses therein, we now use CRE throughout the revised manuscript to avoid confusion. CRE is actually near symmetry at 27 E.

4. L95-96: It's not the trend in R, but rather ΔR, right?

Yes! Thanks for catching this. It has been corrected in the revised manuscript.

5. I'm not a big fan of Fig. 1a. Longitude as color, what a strange choice, but I've convinced myself to accept the intent to show that flux asymmetry and ocean fraction symmetry have a monotonic relationship which is a recurring theme in the paper. But Fig. 1b? Why keep showing ΔO? Why not show longitude of ΔR=0 vs year?

Thanks for the suggestion. In consideration of all three reviewers' comments, we have changed how the E-W symmetry, as well as its robustness, are presented in the revised Fig. 1. As a result, we now show longitude as the x-axis and ΔO as colors.

6. Figure 3 is also curious. ΔR and longitude are never shown on the same plot, as in "what is the longitude where ΔR=0?". It's just a convoluted way of showing things with the choices made for Fig. 3.

We fully agree. In consideration of all three reviewers' comments, Fig. 3 has been fully reconstructed. In the effort to streamline the way the E-W symmetry is presented, we now only show ΔR at 27 E for the E-W symmetry.

7. Why are the authors showing the sum of the hemispheric values (the numbers in the neighborhood of 200 Wm-2) in extended Figures 5 and 8?

These are simply shown to indicate the total SW reflection of the Earth for a particular season, nothing more. This is specified in the caption to avoid confusion.

8. L210, to be exact these are equatorial daytime crossing times.

Thanks. Sentence reworded.

9. L218, please cite the primary ISCCP literature for 3.6 and 23 and not Diamond et al. which is a secondary reference.

Thanks for catching this. Rossow & Schiffer (1999) is now cited in the revised manuscript.

10. In the description of CERES data, I am sure the authors are aware that by going up to December 2024 in EBAF, NOAA-20 satellite data are also involved.

Yes. We have now added this information in the Methods. We also note that there is an apparent offset in between Terra+Aqua and NOAA-20 record in the FBCT dataset, which prevents the use of FBCT beyond 2022.

"L250-252: This data is derived from measurements of SW radiance between 0.3-5 μm from CERES instruments onboard the Terra, Aqua, and NOAA-20 satellites[14–16], and these records have been synced in the EBAF product."

"L257-258: There is an apparent offset in TOA SW fluxes between those measured from NOAA-20 and those measured from Terra+Aqua, preventing us from using the FBCT product beyond 2022."

11. In the equations used to derive Ratm and Rsfc isn't "S" and FTOA_down the same thing?

For EBAF, yes.

For FBCT, because the FBCT product itself doesn't provide surface flux calculations, we had to use EBAF calculated surface fluxes. Since we are calculating transmissivity, absorptivity, and reflectivity (i.e., relative measures) of the atmosphere and the surface, it is important to use a consistent dataset between the sfc and toa fluxes, thus, we use EBAF's toa fluxes when decomposing Ratm and Rsfc in FBCT. In other words, we are estimating transmissivity, absorptivity, and reflectivity only from the EBAF product, and then applied to 'S' from either FBCT and EBAF products. We have added this information to the revised manuscript in the Methods.

"L291-293: For the EBAF decomposition, S and $F\downarrow$ TOA are identical. To ensure consistency with the calculated surface fluxes that are only available from EBAF, we use $F\downarrow$ TOA from EBAF when calculating relative measures in the FBCT decomposition."

12. Why were only the particular 5 climate models selected? Were these the only ones that had performed all the experiments the authors wanted to include?

Yes, they were selected based on the rationale that we want to represent both the variability in model architecture and the variability in climate scenarios. We first group the CMIP6 models into 14 families based on Kuma et al. (2023, JAMES). We then selected 8 of the 14 families that have an Earth System Model (ESM; ocean coupling is a critical part of understanding the symmetry). The IITM-ESM family was excluded due to the lack of the 'siconc' (sea ice) variable. Within each of the 8 families, we then select models that participated in both historical and ssp3-7.0 experiments with a preference given to the model that also participated in the GeoMIP (specifically G6sulfur) experiments. This yields a total of 8 models, one from each family. During the revision, we realized that the ESGF node we were using didn't archive the full range of variables and experiments. After going through all available nodes, we increased the total number of models from 5 to 8 in the revised manuscript. We have now added this model selection rationale and details in the revised manuscript.

"L310-318: We obtained simulation outputs from eight state-of-the-art coupled Earth system models that participated in the Coupled Model Intercomparison Project Phase 6 (CMIP6) \cite{Eyring16} historical experiment, the Scenario Model Intercomparison Project (ScenarioMIP) \cite{ONeill16}, and the Geoengineering Model Intercomparison Project Phase 6 (GeoMIP6) \cite{Kravitz15}. The models were selected to maximize the representation of variabilities in both model architecture and climate scenarios. To do this, we first grouped CMIP6 models into 14 families based on Figure 2 in Kuma et al. (2023) \cite{Kuma23}. We then selected 8 of out the 14 families that have an Earth system model, since ocean coupling is a critical part of Earth albedo symmetries. The IITM-ESM family was excluded due to the lack of the sea ice (`siconc') variable. Within each of the 8 families, we then selected models that participated in both historical and SSP3-7.0 experiments with a preference given to the model that also participated in the G6sulfur experiment (as part of GeoMIP)."

Referee #3:

Review of: "Earth's albedo symmetry at 28° E", Zhang et al.

The authors report an interesting east-west hemispheric symmetry of albedo contributions by the surface, clouds, and the clear-sky atmosphere, coinciding with a symmetry of open ocean fraction, located at the 28° E meridian that hardly varies over the CERES observational record. This intriguing result is proposed to provide a benchmark with which climate models can be compared for realism and to gain understanding of processes driving energy balance and redistribution by ocean and atmospheric circulation. This result is novel; east-west hemispheric symmetries have not yet been investigated or demonstrated, and the newly found symmetries comprising more transient, dynamic

climate system components (atmospheric circulation, clouds, and sea ice) and more fixed components (land/ocean fraction) is fundamentally interesting.

Overall, the manuscript is well-written, concise, and minimal, effectively delivering results from observational and climate simulation analyses. The abstract summarizes the finding effectively and concisely, and the introduction section nicely and efficiently introduces the work. The statistical and calculation methods used are sufficient to present the finding and compare it with climate models in a consistent framework. The data on which it is based are sufficiently robust and reliable, having provided the basis for most observational studies of Earth's radiation balance. Its graphics are high-quality and convey a good amount of interesting information in space-efficient but very readable ways.

My primary concern is about the justification of the finding's significance to climate studies, and the level of confidence in its significance. While I believe that the finding warrants publication and can provide insights into Earth's climate system, the manuscript could be improved by placing its findings in the context of existing work around spatial energetic contrasts like the observed NH-SH albedo symmetry. This also concerns the effectiveness of the manuscript's conclusions section, which could benefit from this comparison so as to not overstate the significance of the findings, and to leave open the possibility that – just like the NH-SH albedo symmetry – the finding may be dependent on the time period considered, and may be affected by emerging changes in Earth's radiation balance beyond the period studied in the manuscript. Aside from this weakness, I believe the findings are significant, and the manuscript could be suitable for publication. Please see below for comments and suggestions that could help improve it.

Sincerely,

Aiden Jönsson

We thank the reviewer for these kind words and insightful comments!

Major comment:

The authors introduce the topic by contrasting it to the NH-SH albedo symmetry (e.g. Stevens and Schwartz, 2012; Voigt et al., 2013; Stephens et al., 2015), referring to the recent publication suggesting that it may be breaking (Loeb et al., 2025), and demonstrating in the results how the E-W symmetry around 28° E persists strongly through the observational record. However, the period (through December 2022) on which this manuscript bases its finding coincides with the period in which NH-SH albedo symmetry is observed in the literature; Loeb et al. (2025) finds that it is primarily after this period that emerging asymmetry can be seen. This might beg the question as to whether or how soon we might expect a change in the reported E-W symmetry, which is not really explored in the manuscript. Extended Data Fig. 7 suggests an emerging trend in cloud asymmetries around 28° E. As the authors find about the E-W symmetry in different climate states in the model comparison section, the N-S albedo symmetry has been projected to change substantially as well (e.g. Rugenstein & Hakuba, 2023; Diamond et al, 2022).

Have the authors looked at the evolution of the E-W symmetry beyond 2022 to ensure that their results are not affected by the choice of study period? These data are available in CERES EBAF ed 4.2.1; is there a practical reason for not using data beyond 2022? The introduction frames an emerging NH-SH asymmetry by citing Loeb et al. (2025) and it is not certain that the E-W symmetry around 28° E is more robust or persistent; ensuring that the text reflects this caveat of the NH-SH albedo symmetry's evolution (perhaps best in the Discussion & conclusions section) could help the reader interpret both symmetries, and leave room to interpret the authors' findings in light of Loeb et al. (2025)'s findings.

We thank the reviewer for these comments, which have motivated us to explore further the persistence of the E-W symmetry and its connection to the N-S symmetry, with a special focus on recent warming records. In light of these comments, we have made the following changes in the revised manuscript:

1. We now extend our analysis to the end 2025, which only became available after the original manuscript was submitted. We note that only the EBAF TOA fluxes are available until the end of 2025, whereas the calculated fluxes at the surface are not, therefore, the EBAF record from 2001-2024 (full year inclusive) is used for the majority of the analysis for decomposition purposes, except when we show the most recent trends in albedo symmetries. Also worth noting is that CERES-measured SW fluxes beyond 2022 are obtained from the NOAA-20 satellite, whose values have been synced with the Terra+Aqua record in the EBAF product, but not in the FBCT product (**Fig. R2**). This prevents us from using the FBCT product beyond 2022. In a nutshell, the majority of the analysis uses EBAF 2001-2024; for cloud type decomposition, FBCT 2003-2022 is used, and for recent trends in ΔR, EBAF 2001-2025 is used. All results and discussions have been updated in the revised manuscript.

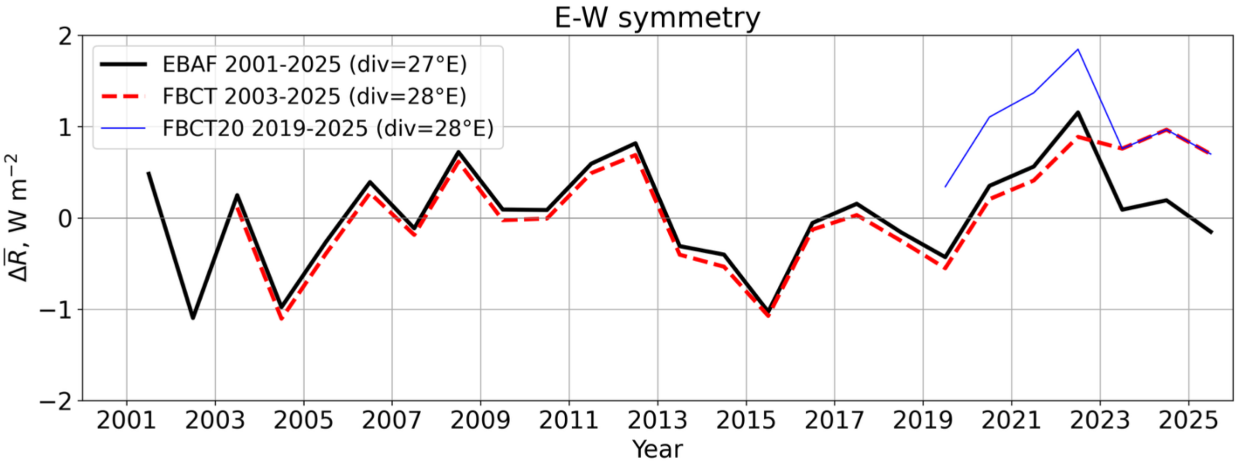

Figure R2. As in Fig. 1c, but based on different CERES products. E-W symmetry is identified at 27 E in EBAF and 28 E in FBCT.

"L277-280: In a nutshell, the EBAF product is used for the majority of the study for its longer record and more complete diurnal-filling, and we use the full record from 2001 to 2025 when assessing the most recent trends in hemispheric albedo trends. The 2001-2024 record is used when TOA flux

decomposition is performed. The record is further limited to 2003-2022 when cloud type decomposition is involved."

2. We now include dedicated discussion on the robustness or persistence of the E-W symmetry and its connection to the persistence of the N-S symmetry throughout the revised manuscript, with reconstructed Figs. 1-4 and Extended Data Fig. 5 and 8.

3. With the updated EBAF record to the end of 2025, we report an intriguing observation that despite the record-breaking temperatures in 2025, the N-S symmetry appears to reverse course back toward symmetry, an update to the finding in Loeb et al. (2025, PNAS) (see new Fig. 1c and Extended Data Fig. 8). The 2001-2025 trend departing from N-S symmetry remains statistically significant (at 95% confidence interval), though.

Minor comments:

L44-48; Figure 1: I am not sure I understand how the variability in the longitude of E-W symmetry depicted in Fig. 1b is consistent with the claim of the 28° E symmetry meridian's robustness. As I interpret it, this figure shows that the average annual mean E-W difference around 28° E is little (and consequently the average of meridians landing at 28° E). However, the variability in symmetry meridians is therefore seemingly very large. Perhaps the manuscript could benefit from a supplementary figure with a PDF of symmetry meridians in the observational record, and the corresponding ΔR around 28° (as in Fig. 3b but replacing ΔO with ΔR), to judge the robustness of this claim.

Indeed, if only one year is used to identify the E-W symmetry, the resulting symmetry-meridian can range from 22 to 33 degrees, with the median and mean close to 27 E. Importantly, at the decadal timescale, the E-W symmetry is robustly identified at 27 E in EBAF (28 E in FBCT). We realize that the original Figs. 1b-e weren't the best way to convey the robustness of the E-W symmetry so we therefore constructed a new Fig. 1b to illustrate this point.

"L62-71: Unlike the N-S symmetry in which the equator is the apparent and only divide for the two hemispheres, there is no such pre-defined divide for the E-W symmetry. In other words, for any given time period, one can try to identify a meridian that separates the Earth into two hemispheres with the same R. The question, then, becomes how robust is the E-W symmetry at 27o E and is it persistent over time? In order to address this question, we identify the E-W albedo symmetry as a function of consecutive year(s) being averaged using the 2001-2025 (inclusive) EBAF record (Fig. 1b). For 1-year averaging, the identified E-W symmetries span ~10 degrees in longitude between 20-30o E (colored circles). Beyond 1-year but shorter than 10-year averaging, an oscillating pattern around 27o E is evident, hinting at an interannual variability in E-W symmetry that might be tied to some multi-year oscillation phenomena (e.g., ENSO; discussed later). At the decadal timescale that encompasses both phases of this oscillation, the E-W symmetry is robustly identified at 27o E. Although variability persists for averaging periods shorter than 10 years, the mean and median are consistently confined between 27oE and 28oE (Fig. 1b)."

L43: The interannual variability in ΔR around 28° E (the variation at 12 months averaging in Fig. 1c) compares reasonably well in magnitude to the interannual variability of the NH-SH symmetry (e.g. ±0.28 W/m2 reported in Datseris and Stevens, 2021; ±0.23 W/m2 reported in Jönsson and Bender, 2022), although it is slightly larger. This is what I assume manifests in the spread in meridians of symmetry in Fig. 1b. Stating this comparison in reported numbers here in the text could help the reader better judge the robustness of the newly found 28° E symmetry by placing it in the context of another symmetry that has been investigated in a number of studies.

Great suggestion! We now state the interannual variability of the E-W symmetry (updated to be ±0.24 W/m2 using EBAF2001-2024) in the context of the reported N-S symmetry interannual variabilities in the revised manuscript.

"L71-73: We observe similar magnitudes of interannual variability in ΔR between the E-W symmetry (at 27 E; ±0.24 W m^{-2}) and the N-S symmetry (at the equator; ±0.20 W m^{-2}) \cite{Datseris21,Jonsson22}) (Fig. \ref{f1}c)."

L53-55, Figure 1a: Although the hemispheric differences in Rcld and Rclr at 28° E are small, there seem to be a few latitudes to the west where symmetry between these components are even stronger, only slightly impacting ΔR. It is hard to tell how much ΔR differs around these meridians, with the scales used in Fig. 1a and with the configuration of 1b; how much does it vary in this region? Since a point is made to call this a triple symmetry, it may be worth noting, while still emphasizing the point that the fact that it occurs near ΔO symmetry is striking.

This is a good point. **Fig. R3** below zooms into this region. The meridian at which R, Rcld (CRE), and Rclr are closest to each other appears at 25 E, at which R symmetry is broken. In the revised manuscript, the "triple symmetry" is now quantitatively stated as within ±0.5 W m2. *"L59-61: In a striking way, symmetries in Rclr and CRE both emerge at approximately 27 E, where the ice-free-ocean fraction is almost identical between the two hemispheres, revealing a unique "triple-symmetry" (±0.5 W m^{-2}; Fig. 1a) that is distinct from the N-S albedo symmetry."*

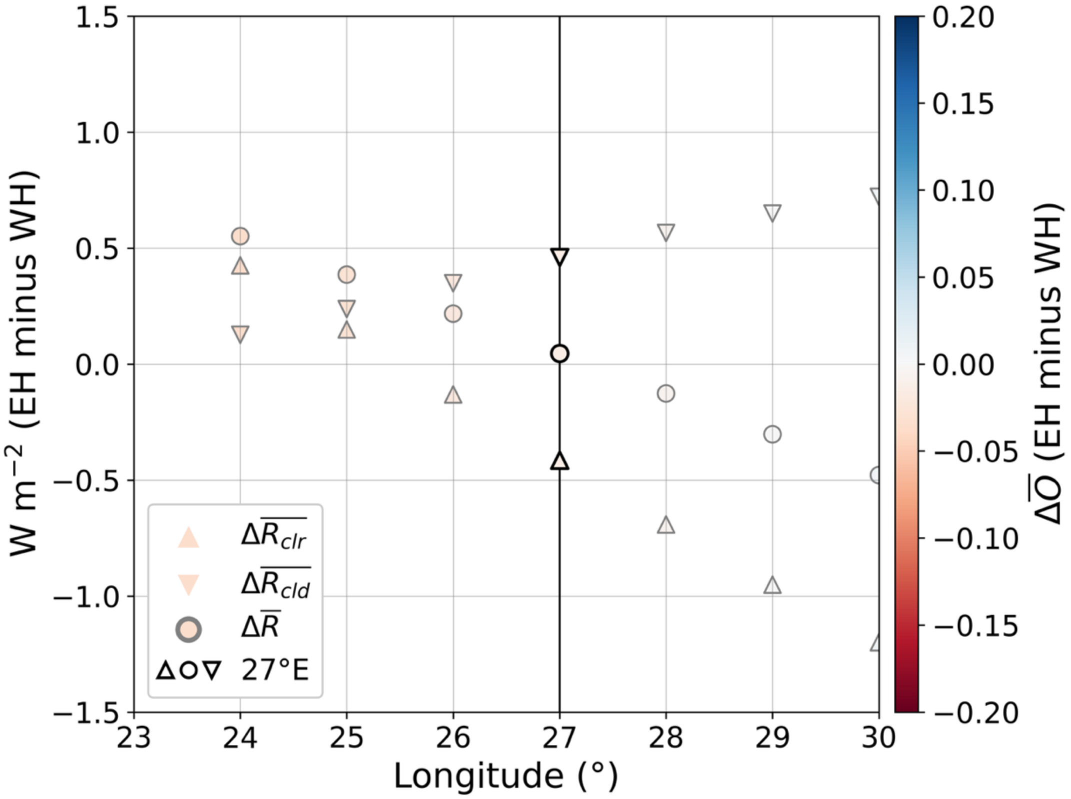

Figure R3. As in Fig. 1a, but zoomed into longitudes between 23E and 30E.

Figure 3: Something seems off with the figure, or I am misunderstanding something; the caption refers to ΔR for (b), but the x axis is labeled ΔO. However, if it is ΔO, I do not understand how these values (spanning -0.2 to +0.2) are consistent with the values plotted in (a) (spanning -0.08 to +0.02). Are they both showing the E-W difference around 28° E along the x axis? If they are different, I suggest that the caption be adjusted for clarity.

Sorry for the confusion, the caption for (b) should have been 'ΔO'. These ΔO values in (b) actually represent ΔO at each simulation's own E-W symmetry, the point being that most models not only miss the symmetry at 27 E, but also miss the observed ΔR-ΔO relationship at symmetry.

In light of comments and similar confusions from the other reviewers on this figure, we have reconstructed Fig. 3 to be consistent with the streamlined storyline in the main text, avoiding a convoluted presentation of the departure from symmetry in the models. In the revised manuscript and the new Fig. 4, the E-W symmetry is always referring to the divide at 27 E.

Figure 3: The authors use single-realization model output to compare the observed E-W symmetry with, and do not characterize the variability in the E-W asymmetry around 28° E in these realizations (such as interannual, anomalies etc.). It could be helpful to include error bars (e.g. standard deviations in annual means) in the model mean asymmetries, because the ability or inability of models to capture the persistence that the authors found in the observed E-W symmetry would be an interesting finding, especially if it is suggested to benchmark models with. I am curious how it

compares to the overestimated variability seen in modeled NH-SH albedo asymmetries across CMIP6 (Fig. 7 of Jönsson & Bender, 2023), which may tell us something about misrepresented processes in the models.

Great suggestion! We now have included error bars for these model realizations, defined as the 95% confidence interval based on interannual variability assuming a red noise process (same way as it is defined in the CERES record). Most models show similar variabilities as that in the CERES record, except for GISS-E2-1-H and CESM2-WACCM. The largest variability is found in CESM2-WACCM's pre-industrial period. Comparing Jönsson & Bender (2022) and the new Fig. 3b, we learned that the degree of persistence in the N-S asymmetry, for most models, is comparable to that in the E-W asymmetry. However, this is not true for the above-mentioned models, in which interannual variability in the E-W asymmetry is much larger than that in the N-S asymmetry.

"L184-186: In general, CMIP6 models show ΔR persistence over the examined time periods (error bars in Fig. 4a) comparable to that seen in the observed albedo symmetries (gray shading), except for GISS-E2-1-H and CESM2-WACCM \cite{Jonsson22}. Notably, CESM2-1 WACCM exhibits particularly large interannual variability in its pre-industrial simulation."

L162-165: The authors find a robust correlation between the ENSO phase and the E-W asymmetry around 28° E, which I find to be the strongest and most convincing argument that the E-W symmetry could be related to or even maintained by large-scale atmospheric circulation. The authors comment on the possibility that the E-W asymmetry in high vs. low clouds could be related to this, which is a good lead for future studies and can point to a possible modulating mechanism. This is also reminiscent of the finding that the most extreme anomalies in the NH-SH albedo symmetry seem to reflect ENSO phase (Jönsson & Bender, 2023), which is an interesting facet of the results. The authors noted the positioning of the EH over the Western Pacific Warm Pool, which is certainly striking; I am wondering if the authors reflected on the relevance of the other rising branch of Walker circulation typically to the east of Central America, and how this affects? Is it that this is a primarily intrahemispheric variability in the WH where the associated high and low cloud variabilities compensate for each other, leaving little effect on the E-W symmetry? While not as prominent as the Pacific branch, it is curious how the EH dominance in high clouds/WH dominance in low clouds manifests considering they both have similar components in overturning (though with different spatial extents), and that ENSO/Walker circulation variability inevitably also alters cloud albedo outside of the Pacific.

These are excellent points! We greatly appreciate them! We have incorporated these comments into the discussion, particularly in the discussion around the Walker circulation. We also put these discussions in the context that a similar connection between ENSO and the N-S symmetry have been made before (Jönsson & Bender, 2022). Motivated by these comments, we now state that shifts in ENSO phases not only affect the rising branch in the Pacific, but also concurrently move the weaker rising branch between western Atlantic and eastern Africa. The 27 E and -153 W meridians are uniquely located at the boundaries of these rising branches during the El Niño phase. Therefore, we

think both rising branches of the Walker circulation contribute to this correlation between ENSO and the E-W symmetry.

“L202-215: We find a strong correlation between ENSO and the interannual variability of the observed E-W hemispheric albedo symmetry at 27o E (Fig. 5). Specifically, the interannual variability of the E-W symmetry is negatively correlated with the Oceanic Niño Index (ONI), an indicator of ENSO phase (see Methods), with a statistically significant correlation coefficient of -0.69 (p-value = 10-4). At the rising branches of the Walker circulation, strong convective ascent leads to expansive, bright anvil clouds capped by the tropopause, contributing substantially to TOA SW reflection. From La Niña to El Niño years, these rising branches, and the corresponding subsiding branches, shift zonally, reshaping cloud and precipitation patterns between E-W hemispheres. This shift in the Walker circulation could trigger cascading, potentially cross-equatorial changes in cloud distribution through coupling with meridional overturning circulations. Indeed, the most extreme interannual anomalies in the N-S symmetry have been linked to ENSO phases \cite{Jonsson22}. Our analysis of the N-S symmetry suggests a weaker and insignificant correlation between ENSO variability and the interannual variability of the N-S symmetry (r=-0.33, p=0.11). This is consistent with the fact that ENSO is largely a mode of variability in the zonal direction. That the observed hemispheric albedo symmetries exhibit different degrees of connection to ENSO suggests they essentially capture the “pulse” of the same climate system in distinct ways. Therefore, potential alteration to atmospheric overturning circulations such as the Walker circulation under climate change[38, 39] could lead to a breakdown of Earth’s albedo symmetries.”

Response to reviews of "Earth's albedo symmetry at 27°E" by J. Zhang et al.

We appreciate the kind words and additional comments from the reviewers. Here, we provide specific point-by-point responses to each comment are contained below, with the reviewers' comments provided in **blue** and our responses in **black**. Changes to the manuscript made in response to the reviewer are provided in ***red italics***.

We have also made changes according to the editor's request and unsolicited updates and changes to the manuscript for clarification and readability, as captured in a tracked changed document.

Referee #1 (Remarks to the Author):

The authors responded to my comments well and I do not have further comments on the new version.

Referee #2 (Remarks to the Author):

I find the revised manuscript a vastly improved version of the original submission and recommend its publication. While I'm not sure this work will have enduring influence, it conveys an intriguing finding that will trigger debates within the ERB community immediately. Here are some additional comments on this version:
-- I'm very satisfied with the revised figure set. It's simpler, cleaner, and more intuitive.
We appreciate the kind words from the reviewer.

-- I'm glad that Rcld was explicitly revealed to be CRE because that created great confusion to me (but why does Rcld still make a cameo since it is CRE that has now been adopted -- see caption of Fig. 1).
We indeed overlooked the Rcld notation in the caption of Fig. 1. We now make sure that 'CRE' is used throughout the manuscript.

-- I agree with the thinking behind the statement that an interhemispheric link is likely more tractable for the E-W rather than N-S symmetry.
Thanks!

-- I see the title of the paper has changed now that the symmetry has shifted to 27°E from 28°E. This shows the perils of being so specific in the title. You add more years to the analysis, longitude may shift again. The analysis dataset is revised/processed you may shift slightly again. I suggest that the authors use a more general title to avoid this pitfall. Like "The Earth also has a longitudinal albedo symmetry"; "Earth's hitherto unknown East-West albedo symmetry". Something like that. Purely a suggestion.
Good point. We fully agree. We have revised the title to be *"Earth's East-West albedo symmetry"*

-- So is this a "triple" symmetry or a "quadruple" symmetry? Because in Fig. 1 the triple symmetry is defined with the three black symbols (caption of Fig. 1a). But a fourth symmetry is also shown, namely the white color indicating equal area of ice-free ocean. The three black symbols only indicate a double symmetry because the quantities are not independent: DCRE=DR-DRclr. The nature of triple symmetry is correctly stated in the concluding section, lines 236-237.
Yes, the "triple symmetry" refers to ocean, Rclr, and CRE, as we stated in the abstract and concluding section. We don't want to call it a "quadruple" symmetry, exactly because, as the

reviewer pointed out, these quantities are not independent. We have revised the wording in caption of Fig. 1a to be consistent with that in lines 236-237 (now lines 315-317).
"Values at 27º E are highlighted with black outlines. An approximate "triple-symmetry" at 27º E, for O, Rclr, and CRE, is a unique feature of the E-W symmetry, distinct from the N-S symmetry."

-- Regarding the transient nature of the E-W symmetry and the parallels with the N-S symmetry: the equator is fixed, but not necessarily the longitude of the E-W symmetry. So when significant deviations from equal reflectance at 27°E start to appear does it simply mean that the longitude of equal reflectance has moved? Transient at a fixed longitude or transient in the sense of a moving longitude of equal reflectance?
Exactly! Both are correct interpretations of the 'transient nature' of the E-W symmetry. A departure from the current observed E-W symmetry at 27E could be either hemispheric difference in SW reflection at 27E is significantly different from zero, or the hemispheric symmetry establishing at a longitude different than 27E. We realized, after reading comments from the first review, using these two interpretations interchangeably can cause confusion and therefore we stick to the first interpretation throughout the revised manuscript.

-- OK, so the models don't have symmetry at 27°E. But do they have symmetry at another longitude? It'd have been interesting to say something specific about that other than their reflectance symmetry is not "necessarily at 27°E" (caption of Fig. 4).
Yes, they do, and we stated this at lines 233-235. In light of the reviewer's comment, we now replace "necessarily at 27°E" with *"ranging from 8º to 131º E".*

-- Why are there no error bars on all values of Fig. 3?
Error bars are now added to all markers in Fig. 3 and Extended Data Fig. 8. We now note in the caption that some error bars are smaller than the size of the markers and thereby not visible.

-- I pointed this out previously, but the authors chose not to do anything about it: Why are hemispheric values added in extended Fig. 4? What is the physical interpretation of the sum? Please re-think this.
These values simply indicate the total climatological SW reflection by Earth for a given season. We agree with the reviewer that they do not add additional physical information to the figure and therefore we have removed them from the revised Extended Data Fig. 4.

-- I like the waterfall plots a lot. Fig. 2b especially conveys a lot of information in a very immediate and concise way. But I wonder whether is problematic to include Extended Fig. 5a which is virtually the same as Fig. 1a of Diamond et al. (only difference is 95.8 vs 95.9 in SH reflectance -- all other numbers are the same). Why show a figure about N-S symmetry? I guess Ext. Fig. 5b is original...
We realized that Fig. 5a is virtually the same as Fig. 1a of Diamond et al. (2024), since we used almost the same period of the CERES FBCT record (offset by 6 months, compared to MD24). Additionally, we follow the same plotting convention for the waterfall chart as in MD24. We included it here only to be complete for our analysis of the N-S symmetry that follows main text Fig. 2, and for ease of comparison with Fig. 2a. This figure was added to the paper after the 1st round of review, as suggested by the other reviewers, to be able to connect the E-W symmetry with the N-S symmetry. We now include a sentence in the caption mentioning the similarity of this to MD24.
"We note that panel (a) is similar to Fig. 1a of Diamond et al. (2024)[6], as we follow the same plotting convention. The slight difference in hemispheric mean R reflects the different analysis period in Diamond et al. (2024)[6], from June 2002 to July 2022."

-- Last line of Fig. 1 caption "are needed".
Thanks! Fixed.

Lazaros Oreopoulos

Referee #3 (Remarks to the Author):

The authors present a finding that the Earth can be divided into eastern and western hemispheres at the 27° E meridian with an intriguing degree of symmetry in clear-sky albedo, (shortwave) cloud radiative effect, and ice-free ocean fraction between them. This symmetry has a similar amount of persistence as the observed Northern-Southern Hemisphere albedo symmetry. The spread in simulated eastern-western hemisphere (a)symmetries around this meridian across GCMs is, like the N-S albedo symmetry, quite high. The E-W symmetry is, which may be expected given the zonal asymmetry of Walker circulation, highly correlated with the ENSO phase, and the authors also show that the E-W asymmetry in high/low cloud cover reflects this.

The revised manuscript feels more relaxed and realistic in its claims about the finding, which significantly improves it. There are a few cases where I reacted to strong language (see minor comments below), but I feel that the framing is honest and does not overclaim the significance of the finding. Moreover, the authors did significant work to situate the finding in the context of the N-S hemispheric albedo symmetry and compare between them, which I believe really helps the work.

Overall, the revised manuscript is very well written and thoroughly describes an interesting new finding certainly worth reporting! I am still somewhat uncertain about the true significance of the finding, but I believe the writing reflects that it is a surprising but open-ended discovery that may or may not lead to deep/useful constraints (just as the N-S albedo symmetry). While I leave it to the editor to decide if it is ultimately fitting for the journal, I am happy to say that the manuscript is essentially ready for publication, but offer a few minor comments below that may help improve it.
We appreciate the kind words and additional comments from the reviewer to help improve the manuscript.

Minor comments:

L9: "... is a clarion call": I reacted to this quite strong wording, as I think there are other things more fitting as a "clarion call" (e.g. the total energy imbalance) when the authors also acknowledge the possibility that the reported discovery may be as coincidental as the N-S albedo symmetry.
Agreed. Wording changed to "…stresses the critical nature of…"

L9, 11, 27, 59: The word "striking" appears many times and it feels a bit like it leads the reader rather than allows them to come to their own conclusions about the finding.
Agreed. We indeed used it more frequently than needed. We have now removed them throughout the manuscript.

L103: "ocean&ice–land": This could do with some reformulation as it feels awkward, perhaps "ocean/ice-land" or "ocean-land" as the cryosphere is not commonly considered land cover.
Thanks! We now use *"ocean/ice-land"*

L198: "... cooler sea surface temperature (SST) branch": I am a bit confused about which branch is being referred to? Of ocean circulation (and if so, where), or something else? If it is the

subsiding branch of the Pacific Walker circulation, then "SST branch" is a bit confusing in atmospheric circulation contexts.
Agreed. The sentence has been re-worded to *"It has been shown that changes in subtropical low-level cloudiness over the subsiding branch of the Pacific Walker circulation can modulate tropical deep convection over its rising branch \cite{Dagan23}, and vice versa \cite{McCulloch25}."*

L228: "... low-degree-of-freedom": I do not really agree that this is a low-degree-of-freedom constraint. There are many ways to arrive at a given planetary albedo, e.g. through differing cloud fractions and optical depths, depending on cloud phase (e.g. Crueger, Schmidt & Stevens, 2023). This "compensating error" feature has been described as a reason for the limited ability of the N-S albedo symmetry to constrain cloud feedbacks (Jönsson et al., 2025) and climate sensitivity (Jönsson & Bender, 2023). While it does remove a few variables compared to the N-S albedo symmetry, the large spread in modeled asymmetries around 27° E hints at a wide range of albedo distributions resulting from differing modeled processes. Would the authors be able to substantiate/elaborate on the claim that it could be a low-degree-of-freedom constraint? If not, I would suggest that this claim also be relaxed.
We fully agree with the reviewer. The word "low" is subjective. We now relaxed this claim by using *"reduced degree-of-freedom constraint"* throughout the manuscript.
